# Biomimetic Studies on the Reactivity of Sulfur-Centered Radicals with Purine Moieties of DNA

**DOI:** 10.3390/biom16050711

**Published:** 2026-05-12

**Authors:** Annalisa Masi, Sebastian Barata-Vallejo, Chryssostomos Chatgilialoglu

**Affiliations:** 1 Istituto di Cristallografia, Consiglio Nazionale delle Ricerche, Via Salaria km 29.300, 00015 Monterotondo, Italy; annalisa.masi@cnr.it; 2 Istituto per la Sintesi Organica e la Fotoreattività, Consiglio Nazionale delle Ricerche, 40129 Bologna, Italy; sebastianbaratavallejo@cnr.it; 3 Departamento de Ciencias Químicas, Facultad de Farmacia y Bioquímica, Universidad de Buenos Aires, Junín 954, Buenos Aires CP 1113, Argentina; 4 Center for Advanced Technologies, Adam Mickiewicz University, 61-614 Poznan, Poland

**Keywords:** DNA damage, sulfur-centered radicals, gamma-radiolysis, purine nucleosides, oligonucleotides, calf-thymus DNA, 5′,8-cyclopurines, 8-oxo-purines, LC-MS/MS

## Abstract

The reaction of the HS^•^/S^•−^ radical (p*K*a ~3.4), generated selectively from H_2_S by γ-irradiated N_2_-flushed aqueous solutions at pH 5, with purine nucleosides (dG or dA), a 10-mer double-stranded oligodeoxynucleotide (ds-ODNs), and calf thymus (ct) DNA was investigated, under various experimental conditions. Concurrent quantification of the four purine 5′,8-cyclo-2′-deoxynucleosides (cPu) and two 8-oxo-7,8-dihydro-2′-deoxypurines (8-oxo-Pu) by LC-MS/MS analysis using isotopomeric internal standards was achieved. The formation of 8-oxo-Pu is several tens of times larger than cPu. Mechanistic schemes for the formation of the two product groups are proposed. Hydrogen atom abstraction from C5′–H by S^•−^ produces the cPu via cyclization of the C5′ radical onto C8, forming a new covalent bond, C5′–C8. The unexpected formation of 8-oxo-Pu should be mechanistically more complex. We propose that an S^•−^ (coupled with H^+^) adds to the base rings, followed by the elimination of HS^−^ to form the corresponding radical cation; subsequent reactions with H_2_O and radical disproportionation with another S^•−^ lead to 8-oxo-Pu. A comparison of S^•−^ with the available literature data for HO^•^ reactivity towards ct-DNA in de-oxygenated aqueous solutions is also presented. Before the present findings, cPu lesions were attributed exclusively to HO^•^ reactivity toward ct-DNA. The reaction of the thiyl radical (HOCH_2_CH_2_S^•^) with ct-DNA was also investigated, yielding results similar to those of S^•−^ obtained under comparable experimental conditions. Our results contributed to a better understanding of DNA damage induced by reactive sulfur species (RSS), particularly the formation of purine lesions and the relative abundance of cPu versus 8-oxo-Pu.

## 1. Introduction

Reactive oxygen and nitrogen species (ROS/RNS) play important roles in biology and cellular signaling [1,2,3,4,5,6]. DNA is vulnerable to excessive levels of these reactive species, which can cause chemical modifications of nucleobases and nucleotides, abasic sites, and DNA strand breaks, as observed in living organisms [7]. Reactive sulfur species (RSS) have also emerged as important mediators of redox regulation and signaling, and their chemistry and biology have been extensively reviewed [8,9,10,11]. Among RSS, hydrogen sulfide (H_2_S) is one of the best-characterized species [8].

H_2_S plays a vital role in physiological processes in the human body. Several enzymes are responsible for producing H_2_S from cysteine, although non-enzymatic pathways from sulfur-containing molecules have also been reported [12,13]. Despite its biological relevance, the radical-based chemistry of H_2_S toward DNA remains poorly understood. Under certain conditions, H_2_S has been implicated in DNA damage, including strand breaks and oxidative damage, through radical-associated and autoxidation-dependent pathways [14,15,16]. Conversely, available evidence indicates a crucial role for H_2_S in DNA damage response and in DNA repair pathways [17,18], helping maintain mitochondrial and nuclear genome stability [19,20]. Moreover, chemical probes have been developed for detecting H_2_S and related RSS in living systems [21]. Overall, these observations place H_2_S at the interface between sulfur redox chemistry and cellular responses that preserve genome integrity. In contrast, the specific reactions of H_2_S-derived sulfur radicals with DNA components remain insufficiently defined.

Alkanethiols (RSH) and the corresponding thiyl radicals (RS^•^), generated in biological systems from low-molecular-weight thiols and protein cysteine residues under oxidative or radiation stress, are increasingly recognized as RSS that can both chemically repair and propagate biomolecular radical damage [22]. In DNA model systems, thiols can quench DNA-centered radicals via H-atom transfer, whereas oxygen competes with this process and promotes radical fixation [23]. Conversely, RS^•^ can abstract H-atoms from carbohydrate C–H bonds [24,25]. In aqueous solutions, rate constants for the reaction of α-hydroxyl alkyl radicals with 2-mercaptoethanol (HOCH_2_CH_2_SH) are ≥10^8^ M^−1^ s^−1^ [26,27], and the resulting alkanethiyl radicals are also able to abstract an H-atom from the sugar moieties [28,29]. The rate constants for the reaction of thiyl radicals from cysteine and a few carbohydrates are reported in the range (1–3) × 10^4^ M^−1^ s^−1^ [28]. The hydrogen abstraction from the methyl moiety of thymidine by cysteamine thiyl radicals occurs with rate constants similar to the hydrogen abstraction from the carbohydrate moieties, providing mechanistic routes to DNA sugar/base radical chemistry under biologically relevant conditions [29,30]. Thus, H-atom donation from thiols to carbon-centered radicals is four orders of magnitude faster than H-atom abstraction from carbohydrate C–H bonds by thiyl radicals.

Regarding the reactivity of RS^•^, it is also worth noting the enzymes ribonucleotide reductase (RNRs), which catalyze the conversion of nucleotides to deoxynucleotides in all organisms, thereby providing the monomeric precursors required for DNA synthesis [31,32,33]. Indeed, a critical step in the catalytic pathway is the reversible H-atom abstraction from C3′–H by the thiyl radical of the cysteine residue.

**Figure 1:**
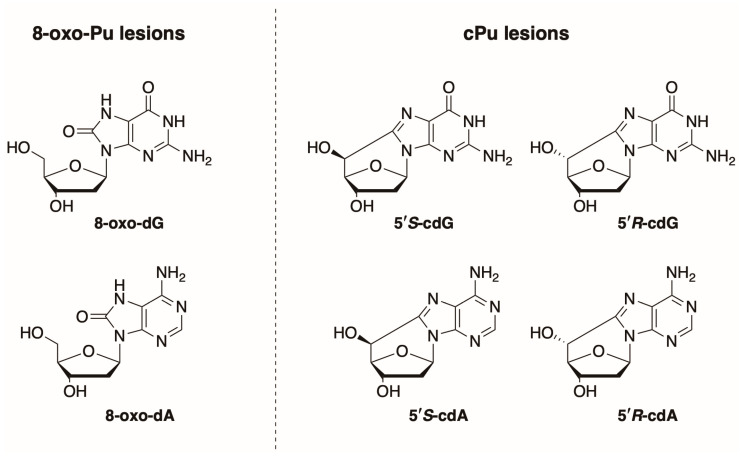
Purine lesions formed after oxidatively induced DNA damage (**left side**) structures of 8-oxo-2′-deoxyguanosine (8-oxo-dG) and 8-oxo-2′-deoxyadenosine (8-oxo-dA) (**right side**) structures of 5′,8-cyclo-2′-deoxyguanosine (cdG) and 5′,8-cyclo-2′-deoxyadenosine (cdA) in their 5′*S* and 5′*R* diastereomeric forms.

A variety of analytical procedures have been developed for the quantification of DNA lesions, among which mass spectrometry-based methods are the most representative [34]. In our group, substantial effort has been directed toward the simultaneous quantification of the six purine lesions shown in Figure 1 [35]. The method was further optimized through model studies of HO^•^ radical reactions with ds-ODNs or commercially available calf thymus (ct) DNA [36,37], and applied in diagnostics of neurodegenerative [38,39,40] or inflammatory bowel diseases [41]. Initially, we planned to identify the cPu lesions resulting from C5′ chemistry (cdA and cdG in their 5′R and 5′S diastereoisomeric forms, as shown in Figure 1) by the action of sulfur-centered radicals. To our surprise, the 8-oxo-Pu were the main lesions. In this work, we apply our protocol to quantify S^•−^ radical-induced cPu and 8-oxo-Pu lesions in purine nucleosides and double-stranded oligodeoxynucleotides (ds-ODNs) in γ-irradiated aqueous solutions. Additionally, the same protocol was used to measure these purine lesions in ct-DNA exposed to either S^•−^ or HOCH_2_CH_2_S^•^ radicals.

## 2. Materials and Methods

### 2.1. Chemicals, Reagents, and Enzymes

Reagents employed for oligonucleotide synthesis were obtained from Sigma-Aldrich, Fluka, and Link Technologies. HPLC-grade acetonitrile, Tris-HCl, NaClO_4_, calf thymus DNA, Na_2_S, and H_3_PO_4_ were obtained from Sigma-Aldrich (St. Louis, MO, USA). Sigma-Aldrich supplied DNase I, DNase II, benzonase (99%), phosphodiesterases I and II, nuclease P1 from *Penicillium citrinum*, deferoxamine mesylate, pentostatin, alkaline phosphatase from bovine intestinal mucosa and BHT. 2′-Deoxyadenosine monohydrate and 2′-deoxyguanosine were purchased from Berry & Associates Inc. (Dexter, MI, USA). Commercial calf thymus DNA was desalted by ethanol precipitation to remove Tris-HCl, NaCl, and EDTA. The DNA pellet was redissolved in 50 mM phosphate buffer (pH 7.2), heated at 90 °C for 5 min, and slowly cooled to room temperature. Isotope-labeled internal standards corresponding to 5′*R*-cdA, 5′*S*-cdA, 5′*R*-cdG, 5′*S*-cdG, 8-oxo-dG, and 8-oxo-dA (see Supporting Information, Figure S3) were synthesized following the procedure described previously [35]. Ultrapure water (18.3 MΩcm) and Milli-Q distilled deionized water were obtained using a Milli-Q purification system (Merck-Millipore, Bedford, OH, USA).

### 2.2. Oligodeoxynucleotides (ODNs) Synthesis and Purification

ODNs were synthesized on a 1 µmol scale by standard DMT/β-cyanoethyl phosphoramidite chemistry on 500 Å CPG supports using an Expedite 8900 DNA synthesizer (Applied Biosystems, Waltham, MA, USA). After synthesis, the DMTr-on ODNs were cleaved from the solid support and deprotected by the two-syringe method using AMA reagent [NH_4_OH (30%)/CH_3_NH_2_ (40%), 1:1] for 10 min at room temperature. The resulting solution was transferred to a sealed vial and heated at 55 °C for 15 min, and the solvent was then removed under reduced pressure in a SpeedVac (Savant Instruments, Inc., Farmingdale, NY, USA).

Crude 5′-DMT-on oligomers were purified and detritylated by RP-HPLC (Grace Vydac C18 column, 5 µm, 50 × 22 mm), further purified by SAX-HPLC (DNA Pac PA-100 column, 5 µm, 22 × 250 mm), desalted on Sep-Pak C18 cartridges, and lyophilized. DNA yields were determined by UV absorbance at 254 nm, and purified ODNs were characterized by MALDI-TOF mass spectrometry (ODN1 and ODN2) (see Table S1 and Figure S1A).

### 2.3. Preparation of Double-Stranded Oligonucleotide Substrates

Complementary oligonucleotide strands were annealed in equimolar amounts in 50 mM sodium phosphate buffer (pH 7.2). Duplex substrates were obtained by heating the strand mixture at 90 °C for 10 min, slowly cooled to room temperature, and analyzed for Tm by UV absorbance at 260 nm using a Cary 100 spectrometer (Agilent, Cernusco sul Naviglio, Italy) equipped with a 1 mL quartz cuvette (1 cm path length). Thermal denaturation profiles were recorded from 20 to 80 °C at a heating rate of 0.3 °C min^−1^. UV melting curves of the 10-mer duplex ODN1/ODN2 are shown in Figure S2A.

### 2.4. γ-Radiolysis Experiments

All solutions of 2′-deoxyadenosine (dA, 0.5 mg/mL), 2′-deoxyguanosine (dG, 0.5 mg/mL), the dG:dA mixture (6:4, total concentration 0.5 mg/mL), ds-ODN1/ODN2 (0.5 and 0.3 mg/mL), and ct-DNA (0.5, 0.3, and 0.1 mg/mL) were prepared in N_2_ saturated water while continuously bubbling N_2_ in the photolysis apparatus. Either sodium sulfide nonahydrate (Na_2_S_•_9H_2_O) in water (400 mM or 30 mM), with pH adjusted to 5 using phosphoric acid (H_3_PO_4_), or 2-mercaptoethanol (30 mM) at native pH (~7), was added. The resulting solutions were transferred to 2 mL glass vials. All additions and transfers were performed under N_2_ atmosphere, degassing the vials. Irradiations were performed at room temperature (22 ± 2 °C) using a Gammacell ^60^Co (220 Nordion Gammacell, Ottawa, ON, Canada) at different absorbed doses (dose rate: 1.87 Gy/min). The exact absorbed radiation dose was determined with the Fricke chemical dosimeter, assuming G(Fe^3+^) = 1.61 μmol J^−1^ [42]. Specifically, the irradiation doses used were 10, 20, and 35 Gy. All irradiation experiments were performed in triplicate, and samples were freeze-dried after irradiation.

### 2.5. Statistical Analysis

All measurements, except for the zero-dose point (single measurement), were performed in triplicate and are reported as mean ± standard deviation (SD), reflecting the variability of the experimental measurements. Dose–response relationships were analyzed by linear regression. Weighted least squares was applied using weights equal to 1/σ^2^, with the intercept constrained to zero. Slopes are reported with their standard errors (SEs), which reflect the uncertainty of the fitted parameter and include the residual variance of the regression.

## 3. Results and Discussion

### 3.1. The Reaction of Sulfhydryl Radical (HS^•^/S^•−^) with Purine Derivatives

#### 3.1.1. Sulfhydryl Radical Generation by γ-Radiolysis

H_2_S is a weak acid (p*K*a 6.98, at 25 °C) (Reaction 1) [8]. H-atom abstraction or one-electron oxidation from either H_2_S or HS^−^ affords the sulfhydryl radical (HS^•^/S^•−^, p*K*a ~3.4) (Reaction 2) [43]. The S^•−^ species, the simplest thiyl radical analog, can abstract a hydrogen [44], add reversibly to carbon–carbon double bonds [45], and react with oxygen to give SO_2_^•−^ [43]. Importantly, S^•−^ adds reversibly to HS^−^ to give HSS^•2−^, with forward and reverse rate constants *k*_3_ = 4.0 × 10^9^ M^−1^ s^−1^ and *k*_−__3_ = 5.1 × 10^5^ s^−1^, and *K*_eq_ = 8000 M^−1^ (Reaction 3) [43]. Based on its reduction potentials, *E*(HSS^•2−^,H^+^/2HS^−^) = +0.69 V vs. NHE at pH 7 and *E*(HSS^−^/HSS^•2−^) ≈ –1.13 V [46], this dimeric radical is a reducing agent for a variety of molecules, including the fast reaction with oxygen (*k* = 4 × 10^8^ M^−1^ s^−1^) to give O_2_^•−^ and HSS^−^.

(R1) H_2_S ⇆ H^+^ + HS^−^

(R2) HS^•^ ⇆ H^+^ + S^•−^

(R3) S^•−^ + HS^−^ ⇆ HSS^•2−^

The bond dissociation enthalpy of H_2_S, *DH*_298_(HS–H), is 91.2 ± 0.1 kcal/mol, which is 3.8 kcal/mol stronger than the S–H bond in methanethiol [*DH*_298_(CH_3_S–H) = 87.4 ± 0.5 kcal/mol] [47]. H_2_S reactivity is therefore expected to be weaker than that of thiols (RSH) with respect to H-atom donation and higher in H-atom abstraction by the S^•−^ than RS^•^ [48]. Indeed, the primary alkyl radical Me_2_C(OH)CH_2_^•^ abstracts H-atoms from HS^−^ with a rate constant 8.3 × 10^4^ M^−1^ s^−1^, which is two orders of magnitude smaller than with RSH [49].

Radiolysis of neutral water produces the reactive species e_aq_^−^, HO^•^, and H^•^ (Reaction 4), along with H^+^ and H_2_O_2_. The values in parentheses are the radiation chemical yields (*G*), expressed in μmol J^−1^ [26,50]. N_2_-flushed aqueous solutions at pH 5 (adjusted with H_3_PO_4_) containing Na_2_S_•_9H_2_O (0.4 M) were the starting point. Under these conditions, the ratio H_2_S/HS^−^ is about 100:1, and the concentration of dissolved molecular H_2_S is ~0.1 M, since the solubility of H_2_S in water is ~4.0 g/L at 20 °C under 1 atm of H_2_S [51]. The e_aq_^−^ is efficiently trapped by ~0.1 M of H_2_S to yield H^•^ atoms (Reaction 5, *k*_5_ = 9.2 × 10^9^ M^−1^ s^−1^ [43]). Thus, H^•^ atoms and HO^•^ radicals account for 55 and 45%, respectively, of the reactive species. Both HO^•^ radicals and H^•^ atoms react readily with hydrogen sulfide at a rate constant of 10^10^ M^−1^ s^−1^ (Reactions 6 and 7) [43]. The resulting HS^•^ has a p*K*_a_ of 3.4 (Reaction 2), and therefore, the S^•−^ and its equilibrium with HSS^•2−^ are the only species at pH 5 (Reaction 3).

(R4) H_2_O + ɣ-irr → e_aq_^−^(0.27), HO^•^(0.28), H^•^(0.062)

(R5) e_aq_^−^ + H_2_S → H^•^ + HS^−^

(R6) H^•^ + H_2_S/HS^−^ → H_2_ + HS^•^

(R7) HO^•^ + H_2_S/HS^−^ → H_2_O + HS^•^

#### 3.1.2. Dose-Dependence of Product Formation in Purine Nucleosides

The reactions of S^•−^ with 2′-deoxyguanosine (dG) and 2′-deoxyadenosine (dA) were studied under standard radiolytic conditions. A total of 200 μL of N_2_-flushed aqueous solutions containing dG_•_H_2_O or dA_•_H_2_O (0.5 mg/mL, which corresponds to ~1.8 mM) and Na_2_S_•_9H_2_O (0.4 M) at pH 5 (adjusted with H_3_PO_4_) were irradiated (in triplicate) at room temperature under steady-state conditions at different doses (0, 10, 20, and 35 Gy) with a dose rate of 1.75 Gy min^−1^. The samples were lyophilized after the irradiation experiments. Product quantification was performed in two independent steps. First, the sample was analyzed on an HPLC-UV system coupled with a sample collector. During this initial cleanup step, unmodified nucleosides were quantified by absorbance at 260 nm, and fractions were collected and pooled during the time windows when modified nucleosides eluted. The concentrated fractions containing the modified nucleosides were then injected into LC-MS/MS for independent analysis and quantification. The use of isotopically labeled lesions (Figure S3) ensures the reproducibility and recovery yield of the quantification protocol at levels generally accepted for reliability [35]. The calibration curves for lesion quantification and the list of MRM transitions used are reported in Figure S4 and Table S2, respectively.

**Figure 2:**
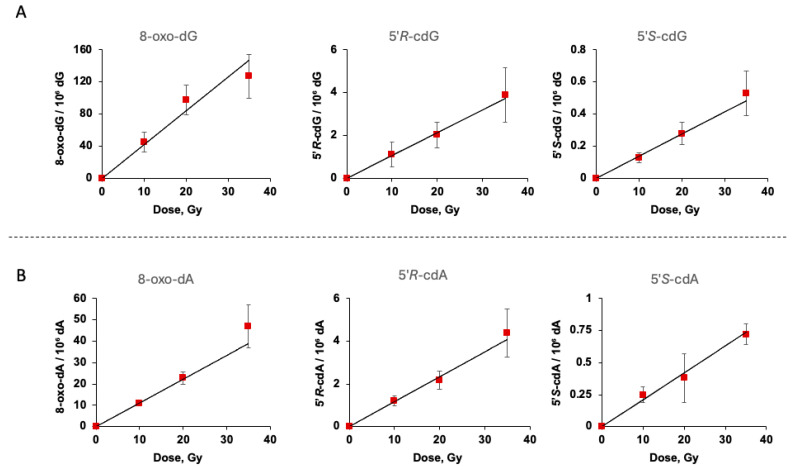
(**A**) Formation of 8-oxo-dG, 5′*R*-cdG, and 5′*S*-cdG from γ-radiolysis of N_2_-flushed aqueous solutions containing dG (1.86 mM) and Na_2_S_•_9H_2_O (0.4 M) at pH 5; the samples were exposed to 10, 20, and 35 Gy doses, and the values represent the mean per 10^6^ dG ± SD of *n* = 3 independent experiments. (**B**) Formation of 8-oxo-dA, 5′*R*-cdA, and 5′*S*-cdA from γ-radiolysis of N_2_-flushed aqueous solutions containing dA (1.75 mM) and Na_2_S_•_9H_2_O (0.4 M) at pH 5; the samples were exposed to 10, 20, and 35 Gy doses, and the values represent the mean per 10^6^ dA ± SD of *n* = 3 independent experiments. Dose rate of 1.75 Gy/min.

The γ-irradiation of dG induced the formation of 8-oxo-dG, 5′*R*-cdG, and 5′*S*-cdG in all samples (Figure 2A and Table S3), whereas the γ-irradiation of dA induced the formation of 8-oxo-dA, 5′*R*-cdA, and 5′*S*-cdA in all samples (Figure 2B and Table S4). The slopes of the lines from Figure 2 represent the quantities of products/10^6^ dG or dA per Gy of irradiation. In Table 1, the products are reported as numbers using the units of 10^7^ dG/Gy or 10^7^ dA/Gy (entries 1 and 2, respectively). From Table 1 can be calculated the ratio 8-oxo-dG/cdG = 35 with a diastereoisomeric ratios 5′*R* /5′*S* = 7.6, and 8-oxo-dA/cdA = 8.1 with 5′*R*/5′*S* = 5.5 (entries 1 and 2, respectively). Compared to the reactions between dG and dA, the 8-oxo-dG is 3.8 times greater than 8-oxo-dA, whereas the formation of cdG and cdA is similar.

**Figure 3:**
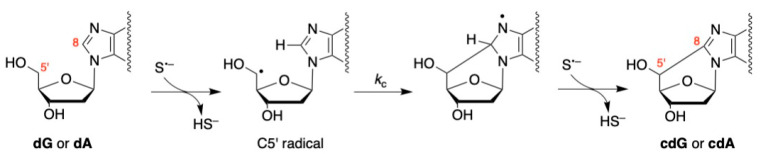
Proposed mechanistic scheme for the formation of cdG and cdA from the reaction of S^•−^ with dG and dA, respectively, via H5′-abstraction by S^•−^, followed by C5′ radical cyclization and radical disproportionation with another S^•−^ species.

Based on the experimental conditions discussed above, it must be underlined that H_2_S/HS^−^ efficiently quenches all hydroxyl radicals (HO^•^) and the equilibrium between S^•−^ and HSS^•2−^ (Reaction 3) regulates the reaction outcome. The proposed reaction mechanisms for the formation of 5′,8-cyclopurines are outlined in Figure 3 and are based on the well-understood reactions of dG and dA with HO^•^ radicals. That is, the HS^•^/S^•−^ radical abstracts the H5′–atom followed by C5′ radical cyclization and presumably radical disproportionation with another S^•−^ for the re-aromatization of the bases, yielding cdG or cdA. The rate constants of C5′ radical cyclization are reported to be 6.9 × 10^5^ and 1.6 × 10^5^ s^−1^ at room temperature for dG and dA, respectively [52,53,54]. It is gratifying to see that the estimated diastereomeric ratio 5′*R*-cdG/5′*S*-cdG = 7.6 and 5′*R*-cdA/5′*S*-cdA = 5.5 from Table 1 is similar to those reported in the isolated products in the reaction of dG and dA with HO^•^ radicals, i.e., 5′*R*-cdG/5′*S*-cdG = 8.3 and 5′*R*-cdA/5′*S*-cdA = 6, for which the diastereomeric outcome has been rationalized by favorable hydrogen-bonding interactions in the *pro*-(5′*R*) conformation [53,54,55]. It is worth mentioning that the formation of C5′ radical is a reversible process (Reaction 8). Therefore, the C5′ radical cyclization competes with H-atom abstraction from H_2_S. Being the concentration of H_2_S ~0.1 M and *k*_8_ close to 10^4^ M^−1^ s^−1^ [29,49], the radical cyclization is at least two orders of magnitude higher than the reverse H-atom abstraction.

(R8) $$S^{•-}+{C5}^{\prime }-H\overset{+H^{+}}{\underset{-H^{+}}{⇌}}H_{2}S+{C5}^{\prime •}$$

**Figure 4:**
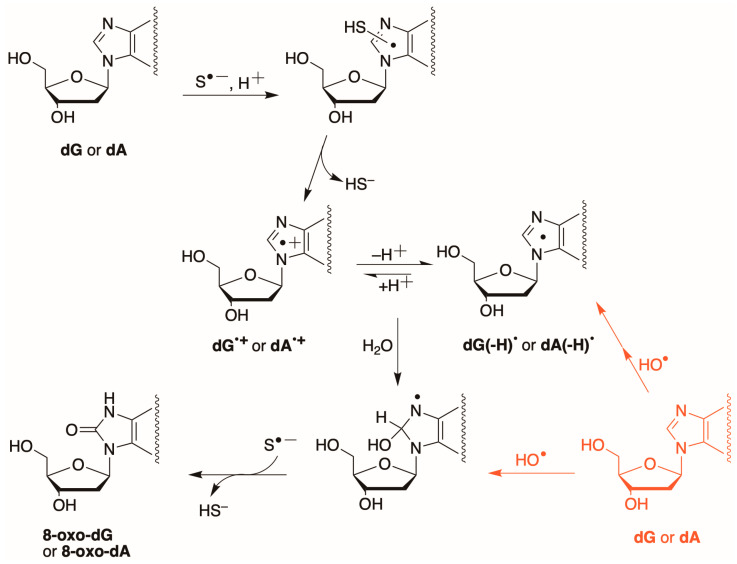
Proposed mechanistic scheme for the formation of 8-oxo-dG and 8-oxo-dA from the reaction of S^•−^ with dG and dA, respectively, via formation of a radical cation, water addition, and radical disproportionation with another S^•−^; the p*K*_a_ values of dG^•+^ and dA^•+^ are 3.9 and 4.2, respectively [60,61]. In red are the two pathways in which the HO^•^ radical with dG or dA generates the 8-oxo-dG or 8-oxo-dA.

On the other hand, the formation of 8-oxo-dG and 8-oxo-dA upon reaction of the HS^•^/S^•−^ radical with dG and dA, respectively, is unexpected. Generally, these products are formed by direct oxidation at C8 positions of bases or by one-electron oxidation followed by reaction with H_2_O [56,57,58]. Considering the reduction potentials of dA (1.42 V) and dG (1.29 V) [59], and the reduction potential of S^•−^, *E*°(S^•−^,H^+^/HS^−^) = +0.91 V [46], neither dA nor dG can be oxidized by outer-sphere electron-transfer (ET) that occurs between chemical species that remain separate and intact. We propose the reaction mechanism reported in Figure 4: addition of HS^•^/S^•−^ radical followed by elimination of HS^−^ to generate the radical cation in purine bases, i.e., an inner-sphere ET that proceeds via a covalent linkage. A driving force for this reaction should be the high hydration energy of HS^−^, which facilitates the inner-sphere ET concerted with the proton transfer. Subsequent reactions with H_2_O [57,58], and radical disproportionation with another S^•−^ for the re-aromatization of the bases, yielding 8-oxo-dG or 8-oxo-dA. The p*K*_a_ values of dG^•+^ and dA^•+^ are 3.9 and 4.2, respectively [60,61,62], and the hydrolysis of deprotonated forms is much less effective. The 3.8 times higher yield of 8-oxo-dG than 8-oxo-dA (Table 1, entries 1 and 2) may be due to the higher reactivity of S^•−^ towards dG. Obviously, this is a mechanistic hypothesis, and alternative mechanisms cannot be excluded based on the present experiments. In Figure 4 (in red), the reactivity of HO^•^ radical with dG [55] and dA [61,63] is also reported for comparison.

Next, we considered the mix of dG and dA. The samples of N_2_-flushed aqueous solutions containing a 0.5 mg/mL dG/dA ratio of 6:4 and Na_2_S_•_9H_2_O (0.4 M) at pH 5 (adjusted with H_3_PO_4_) were irradiated (in triplicate) at room temperature under steady-state conditions at different doses (0, 10, 20, and 35 Gy) with a dose rate of 1.75 Gy min^−1^. The mean values ± SD of 8-oxo-dG, 5′*R*-cdG, 5′*S*-cdG, 8-oxo-dA, 5′*R*-cdA, and 5′*S*-cdA from *n* = 3 independent experiments are reported in Table S5. Figure S5 illustrates the plots of six products/10^6^ dG (or 10^6^ dA) vs. dose (Gy), where the slope of the lines represents the quantities of products formed per Gy of irradiation. Table 1 (entry 3) summarizes the product/10^7^ dG/Gy or product/10^7^ dA/Gy. It is gratifying to see that the similarity of ratios 8-oxo-dG/cdG, 8-oxo-dA/cdA, and 5′*R*/5′*S* in both cdG and cdA, with single nucleoside experiments.

#### 3.1.3. Dose-Dependence of Lesion in ds-ODNs

The reaction of HS^•^/S^•−^ radicals with double-stranded 10-mer oligonucleotide ODN1/ODN2 (T_m_ = 42 °C) was studied:

**ODN1**: 5′-GCG TTTT GCG-3′ **ODN2**: 5′-CGC AAAA CGC-3′

For this purpose, 200 μL of N_2_-flushed aqueous solutions containing ds-ODN (0.5 or 0.3 mg/mL) and Na_2_S_•_9H_2_O (0.4 M) at pH 5 (adjusted with H_3_PO_4_), were irradiated (in triplicate) under steady-state conditions with a dose rate of 1.75 Gy min^−1^ at room temperature. The samples were exposed to doses 10, 20, and 35 Gy and then lyophilized. Each sample was processed using our optimized enzymatic oligonucleotide digestion and LC-MS/MS analysis [35]. Specifically, lesion quantification was performed in two independent steps as described above.

The γ-irradiation caused the formation of six lesions (four cPu and two 8-oxo-Pu) in all samples. Since the sequence of the double-stranded 10-mer is well defined, we calculated that ds-ODN contains 25% dG and 20% dA, which can produce lesions such as 8-oxo-dG, 5′S-cdG, and 5′R-cdG per 10^6^ dG, and 8-oxo-dA, 5′S-cdA, and 5′R-cdA per 10^6^ dA. The average values ± SD of the six lesions from three independent experiments are reported in Tables S6 and S8 for 0.5 mg/mL and 0.3 mg/mL ds-ODN, respectively. In all cases, lesion formation increased with the applied dose within the range of 0–35 Gy. Figures S6 and S7 show the levels of six purine lesions versus dose (Gy), with the slope of each line indicating the quantity of products formed per Gy of irradiation, reported in Table 1 (entries 4 and 5), in units of lesion/10^7^ dG/Gy or lesion/10^7^ dA/Gy.

**Figure 5:**
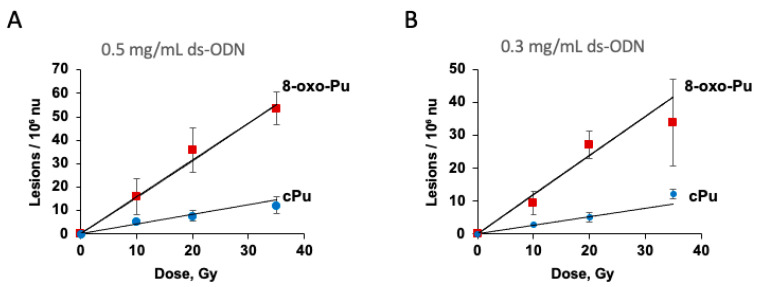
Formation of 8-oxo-Pu and cPu lesions from γ-radiolysis of N_2_-flushed aqueous solutions containing 0.5 mg/mL ds-ODN (**A**) or 0.3 mg/mL ds-ODN (**B**) and Na_2_S_•_9H_2_O (0.4 M) at pH 5. The samples were exposed to 10, 20, and 35 Gy doses. The values represent the mean per 10^6^ nu ± SD, based on *n* = 3 independent experiments. Dose rate of 1.75 Gy/min.

Tables S7 and S9 summarize the combined data for 8-oxo-Pu and cPu per 10^6^ nucleosides for ds-ODN at 0.5 and 0.3 mg/mL, respectively, while Figure 5A,B plot lesions per 10^6^ nucleosides versus dose (Gy). Reducing ds-ODN concentration from 0.5 to 0.3 mg/mL decreases the slopes of 8-oxo-Pu from 15.7 ± 0.6 to 11.9 ± 1.4, and cPu slopes from about 4.3 ± 0.6 to 3.0 ± 0.3 lesions/10^7^ nu/Gy. The 8-oxo-Pu lesions are roughly four times more frequent than cPu lesions. The diastereomeric ratios, 5′*R*-cdG/5′*S*-cdG ~0.5 and 5′*R*-cdA/5′*S*-cdA ~1.5, are similar to those reported previously for ds-ODN with HO^•^ radicals, confirming the reaction mechanism of C5′ radicals regardless of their formation method. [36,37].

#### 3.1.4. Dose-Dependence of Lesion Formation in ct-DNA

The reaction of HS^•^/S^•−^ radicals with ct-DNA was studied under standard radiolytic conditions across three different ct-DNA concentrations. A total of 200 μL of N_2_-flushed aqueous solutions containing ct-DNA (0.5, 0.3, or 0.1 mg/mL) and Na_2_S_•_9H_2_O (0.4 M) at pH 5 (adjusted with H_3_PO_4_) were exposed to 10, 20, and 35 Gy doses (in triplicate) at a steady-state dose rate of 1.87 Gy min^−1^ at room temperature. After irradiation, the samples were lyophilized and analyzed as described earlier. Data processing was similar to previous methods, i.e., (i) the mean values (lesion/10^6^ nu) ± SD of 8-oxo-dG, 8-oxo-dA, 5′*R*-cdG, 5′*S*-cdG, 5′*R*-cdA, and 5′*S*-cdA are reported in Tables S10–S12 for concentrations of 0.5, 0.3, or 0.1 mg/mL of ct-DNA; (ii) the corresponding Figures S8–S10 show plots of lesions/10^6^ nu versus dose (Gy), with slope values reported in Table 2.

Table 2 shows that the six lesion levels (lesions/10^7^ nu/Gy) decrease as the ct-DNA concentration drops, roughly halving from 0.5 to 0.3 mg/mL and decreasing further from 0.3 to 0.1 mg/mL. Table 3 shows the various ratios of the data from Table 2. It is gratifying to see the same ratios 8-oxo-dG/8-oxo-dA, cdG/cdA, 5′*R*/5′*S* of cdG, and 5′*R*/5′*S* of cdA by substantially decreasing the ct-DNA concentration, supporting the quality of our analytical protocol [35], and indicating that no secondary processes are involved.

It is possible to compare the reactivities of S^•−^ and HO^•^ radicals with ct-DNA at a concentration of 0.5 mg/mL. The S^•−^ is generated by γ-radiolysis of N_2_-flushed aqueous solutions containing ca. 0.1 M H_2_S (as described herein), while HO^•^ is generated by γ-radiolysis of N_2_O-saturated solutions [36]. In these experiments, the radiation chemical yields (*G*) of S^•−^ and HO^•^ are similar, *G*(S^•−^) = 0.61 and *G*(HO^•^) = 0.55 μmol J^−1^, the S^•−^ being in equilibrium with HSS^•2−^. Table 4 summarizes the levels of 8-oxo-Pu and cPu as well as the various ratios within each group.

It is worth mentioning that the reaction of HO^•^ radicals with DNA occurs by H-atom abstraction from the 2′-deoxyribose units and by addition to the base moieties, the latter accounting for ~85% of attacks in naked DNA [64]. The order of reactivity of HO^•^ towards the various H-atoms of the 2′-deoxyribose moieties in DNA is widely accepted to be H5′ > H4′ > H3′ ≈ H2′ ≈ H1′, which goes along with the exposure of H-atoms to the solvent [65,66]. It is estimated that ca. 7% of HO^•^ radicals abstract the H5′ in naked DNA [67]. Within the various identified purine lesions in vitro and in vivo, the two 8-oxo-Pu (8-oxo-dG and 8-oxo-dA) are the main ones.

The similarities in reactivity of S^•−^ and HO^•^ radicals with ct-DNA are impressive and unpredictable, based on the available kinetic data, which are a few orders of magnitude smaller for S^•−^. Table 3 shows that 8-oxo-Pu lesions per Gy of irradiation are ~20% higher for S^•−^ than HO^•^, whereas cPu lesions are ~15% lower for S^•−^ than HO^•^. Therefore, the ratio 8-oxo-Pu/cPu is much higher for S^•−^ than for HO^•^, indicating that 8-oxo-Pu lesions are 62.3 times more frequent than cPu lesions with S^•−^. Interestingly, the ratios 8-oxo-dG/8-oxo-dA and cdG/cdA for both reactive species are identical.

**Figure 6:**
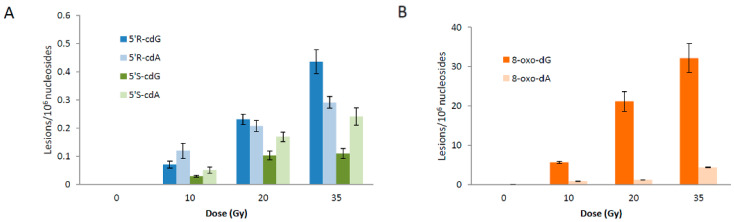
Radiation-induced formation of 5′*R*-cdG, 5′*R*-cdA, 5′*S*cdG, and 5′*S*-cdA lesions (**A**), and 8-oxo-dG and 8-oxo-dA lesions (**B**); 200 μL of N_2_-flushed aqueous solutions containing ct-DNA (0.1 mg/mL) and Na_2_S_•_9H_2_O (30 mM) at pH 5 were irradiated under steady-state conditions with a dose rate of 1.44 Gy min^−1^ at room temperature. The samples were exposed to 10, 20, and 35 Gy doses. The values represent the mean ± SD of *n* = 3 independent experiments.

**Figure 7:**
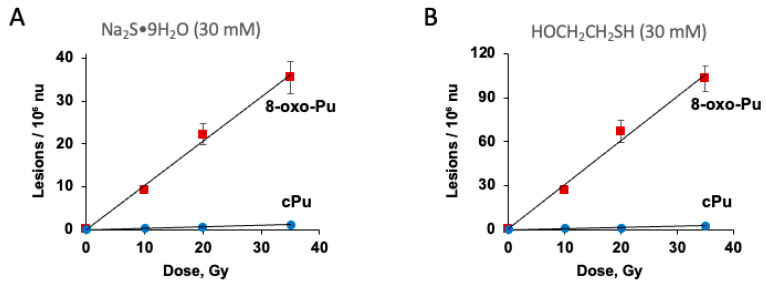
Formation of 8-oxo-Pu and cPu lesions from γ-radiolysis of N_2_-flushed aqueous solutions containing 0.1 mg/mL ct-DNA and Na_2_S_•_9H_2_O (30 mM) at pH 5 (**A**) or HOCH_2_CH_2_SH (30 mM) at pH 7 (**B**). The samples were exposed to 10, 20, and 35 Gy doses. The values represent the mean per 10^6^ nu ± SD, based on *n* = 3 independent experiments. Dose rate of 1.44 Gy/min.

Next, we considered the possibility of decreasing the concentration of H_2_S considerably using 30 mM of Na_2_S_•_9H_2_O and 0.1 mg/mL of ct-DNA. The reaction works quite well. The mean values (lesion/10^6^ nu) ± SD of the six lesions from *n* = 3 independent experiments are illustrated in Figure 6. Figure 6A,B show the growth of four cPu and 8-oxo-Pu lesions, respectively, at 0, 10, 20, and 35 Gy (with data compiled in Table S13). The corresponding Figure S11 shows plots of lesions/10^6^ nu versus dose (Gy), with slope values (levels/10^7^ nu/ Gy) reported in Table 5. Moreover, Figure 7A reports the total amount of 8-oxo-Pu and cPu lesions vs. dose (Gy) (data compiled in Table S14), with slope values of 8-oxo-Pu/10^7^ nu/Gy of 9.3 ± 0.3 and cPu/10^7^ nu/Gy 0.30 ± 0.03.

### 3.2. The Reaction of Thiyl Radical with ctDNA

#### 3.2.1. Thiyl Radical Generation by γ-Radiolysis

The formation of alkanethiyl radicals (RS^•^) is one of the most important reactions in biological systems, e.g., the intracellular concentration of glutathione (GSH) is in the range of 1–10 mM (depending on the cell type) and serves as an H-atom donor in the repair mechanism, producing GS^•^ radical [68].

Pulse radiolysis studies have shown that e_aq_^−^ reacts with alkanethiols (RSH) to produce R^•^ radicals in quantitative yield [69]. In particular, the rate constant for e_aq_^−^ with 2-mercaptoethanol (p*K*_a_ = 9.5) is measured to be 8.2 × 10^9^ M^−1^ s^−1^ (Reaction 9) [70]. The reaction rate constant of HO^•^ radicals with RSH is quite high, with values ≥ l × 10^10^ M^−1^ s^−1^ (Reaction 10) [69]. Steady-state radiation experiments on H-atoms and RSH compounds indicate two modes of attack, a minor one involving H-atom abstraction from the sulfhydryl moiety (Reaction 11) and a major one due to homolytic substitution at the sulfur (Reaction 12) [69]. The rate constant for the reaction of the resulting primary alkyl radical with 2-mercaptoethanol is ≥l × 10^8^ M^−1^ s^−1^ in aqueous solution (Reaction 13) [26,27,71]. Based on the above knowledge, at relatively high RSH concentration, the primary reactive species of γ-radiolysis (e_aq_^−^, HO^•^, and H^•^) are transformed into RS^•^ radicals.

(R9) e_aq_^−^ + HOCH_2_CH_2_SH → HOCH_2_CH_2_^•^ + HS^−^

(R10) HO^•^ + HOCH_2_CH_2_SH → H_2_O + HOCH_2_CH_2_S^•^

(R11) H^•^ + HOCH_2_CH_2_SH → H_2_ + HOCH_2_CH_2_S^•^

(R12) H^•^ + HOCH_2_CH_2_SH → H_2_S + HOCH_2_CH_2_^•^

(R13) HOCH_2_CH_2_^•^ + HOCH_2_CH_2_SH → HOCH_2_CH_3_ + HOCH_2_CH_2_S^•^

#### 3.2.2. Dose-Dependence of Lesion Formation in ct-DNA

**Figure 8:**
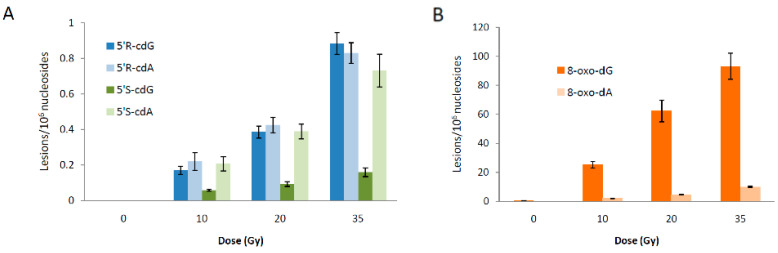
Radiation-induced formation of 5′*R*-cdG, 5′*R*-cdA, 5′*S*cdG, and 5′*S*-cdA lesions (**A**), and 8-oxo-dG and 8-oxo-dA lesions (**B**); 200 μL of N_2_-flushed aqueous solutions containing ct-DNA (0.1 mg/mL) and HOCH_2_CH_2_SH (30 mM) at natural pH were irradiated under steady-state conditions with a dose rate of 1.44 Gy min^−1^ at room temperature. The samples were exposed to 10, 20, and 35 Gy doses. The values represent the mean ± SD from *n* = 3 independent experiments.

The reaction of HOCH_2_CH_2_S^•^ radicals with ct-DNA was examined under specific conditions: 200 μL of N_2_-flushed aqueous solutions containing ct-DNA (0.1 mg/mL) and HOCH_2_CH_2_SH (30 mM) at pH 7 (natural pH). Samples were irradiated (in triplicate) under steady-state conditions at various doses (0, 10, 20, and 35 Gy) with a dose rate of 1.44 Gy min^−1^ at room temperature. After irradiation, the samples were lyophilized. Lesion quantification was performed in two separate steps as described above. The radiation-induced formation of 8-oxo-dG, 5′*S*-cdG, 5′*R*-cdG, 8-oxo-dA, 5′*S*-cdA, and 5′*R*-cdA in ct-DNA is presented in Figure 8, with data compiled in Table S15. It is clear from Figure 8 that the primary reaction products are 8-oxo-dG and oxo-dA. As anticipated, the number of lesions increases with higher doses. Further analysis, by plotting the number of each lesion against radiation dose (Figure S12), allowed determination of lesion levels per 10^7^ nucleotides per Gy from the slopes of the lines, as shown in Table 5. Table 5 indicates that the following ratios can be calculated: 8-oxo-dG/8-oxo-dA = 10.9, 5′*R*-cdG/5′*S*-cdG = 4.2, and 5′*R*-cdA/5′*S*-cdA = 1.1. Additionally, Figure 7B displays the total amount of 8-oxo-Pu and cPu lesions versus dose (Gy) (data from Table S16), with slope values of 8-oxo-Pu/10^7^ nu/Gy at 29.7 ± 2.3 and cPu/10^7^ nu/Gy at 0.73 ± 0.05, indicating that 8-oxo-Pu lesions are 40 times more frequent than cPu lesions.

## 4. Conclusions

In the present biomimetic studies, we demonstrated that the reaction of two sulfur-centered radicals (S^•−^ and HOCH_2_CH_2_S^•^) interacts with the purine moieties (dG or dA) either by hydrogen atom abstraction at the C5′ position to generate the C5′^•^, a precursor of 5′,8-cyclopurine (cPu) lesions, or by addition to the bases, producing 8-oxo-Pu lesions. We propose a mechanistic scheme for the formation of 8-oxo-Pu, but alternative mechanisms cannot be excluded based on the present results.

Using by γ-irradiated N_2_-flushed aqueous solutions containing ct-DNA (0.1 mg/mL) and Na_2_S_•_9H_2_O (30 mM) at pH 5 (a precursor of H_2_S) or HOCH_2_CH_2_SH (30 mM) at pH 7, we obtained similar results. The observed differences can be attributed to thermodynamic variations between sulfur-centered radicals and their corresponding S—H bonds, or to differences in steric effects between the two radicals.

The similarities in reactivity of S^•−^ and HO^•^ radicals with ct-DNA were unexpected. The cPu lesions result from common H-atom abstraction at C5′, although the rate constant is a few orders of magnitude smaller for S^•−^. The formation of 8-oxo-Pu in the case of sulfur-centered radicals needs further studies.

Our work adds a new piece to the DNA damage puzzle to explore further.

## Figures and Tables

**Table 1:** Formation of 8-oxo-dG, 8-oxo-dA, 5′*R*-cdG, 5′*S*-cdG, 5′*R*-cdA, and 5′*S*-cdA from γ-radiolysis of N_2_-flushed aqueous solutions containing nucleosides or ds-ODN (0.5 mg/mL) and Na_2_S_•_9H_2_O (0.4 M) at pH 5. The values 10^7^ dG (or 10^7^ dA) per Gy of γ-irradiation correspond to slopes obtained from linear regression (product formation vs. dose) and are reported as slope ± standard error (SE) ^1^.

Entry	Substrate	8-oxo-dG/ 10^7^ dG/Gy	8-oxo-dA/ 10^7^ dA/Gy	5′*R*-cdG/ 10^7^ dG/Gy	5′*S*-cdG/ 10^7^ dG/Gy	5′*R*-cdA/ 10^7^ dA/Gy	5′*S*-cdA/ 10^7^ dA/Gy
1	dG	42.10 ± 4.05		1.06 ± 0.04	0.14 ± 0.01		
2	dA		11.05 ± 0.40			1.16 ± 0.05	0.21 ± 0.01
3	(dG + dA) ^2^	27.25 ± 2.04	3.02 ± 0.05	0.88 ± 0.01	0.13 ± 0.01	0.30 ± 0.01	0.05 ± 0.03
4	ds-ODN	13.08 ± 0.96	2.39 ± 0.20	0.32 ± 0.05	0.62 ± 0.08	2.24 ± 0.26	1.67 ± 0.14
5	ds-ODN ^3^	10.43 ± 1.33	1.55 ± 0.05	0.32 ± 0.05	0.55 ± 0.07	1.39 ± 0.13	0.93 ± 0.19

^1^ Dose rate of 1.75 Gy/min. ^2^ dG/dA ratio 6:4. ^3^ ds-ODN (0.3 mg/mL).

**Table 2:** The levels (lesions/10^7^ nu/Gy) of 8-oxo-dG, 8-oxo-dA, 5′*R*-cdG, 5′*S*-cdG, 5′*R*-cdA, and 5′*S*-cdA from γ-radiolysis of N_2_-flushed aqueous solutions containing ct-DNA and Na_2_S_•_9H_2_O (0.4 M) at pH 5 (adjusted with H_3_PO_4_). The values correspond to slopes obtained from linear regression (product formation vs. dose) and are reported as slope ± standard error (SE) ^1^.

ct-DNA mg/mL	8-oxo-dG	8-oxo-dA	5′*R*-cdG	5′*S*-cdG	5′*R*-cdA	5′*S*-cdA
0.5	220.8 ± 19.1	28.23 ± 2.81	1.08 ± 0.13	0.43 ± 0.01	0.99 ± 0.03	1.53 ± 0.04
0.3	103.1 ± 14.1	14.11 ± 1.26	0.54 ± 0.05	0.21 ± 0.01	0.52 ± 0.03	0.76 ± 0.02
0.1	40.74 ± 3.28	5.13 ± 0.59	0.21 ± 0.02	0.09 ± 0.01	0.20 ± 0.01	0.29 ± 0.01

^1^ Dose rate of 1.87 Gy/min.

**Table 3:** Various ratios of lesions/10^7^ nu/Gy from γ-radiolysis of N_2_-flushed aqueous solutions containing ct-DNA and Na_2_S_•_9H_2_O (0.4 M) at pH 5 (adjusted with H_3_PO_4_).

ct-DNA mg/mL	8-oxo-dG/8-oxo-dA	cdG/cdA	5′*R*/5′*S* of cdG	5′*R*/5′*S* of cdA
0.5	7.8	0.6	2.5	0.65
0.3	7.3	0.6	2.6	0.68
0.1	7.9	0.6	2.3	0.69

**Table 4:** Comparison of 8-oxo-Pu and cPu lesions from the reactions of S^•−^ and HO^•^ radicals with ct-DNA at a concentration of 0.5 mg/mL ^1^.

Radical	pH	8-oxo-Pu/10^7^ nu/Gy (8-oxo-dG/8-oxo-dA)	cPu/10^7^ nu/Gy (cdG/cdA)	cdG 5′*R/*5′*S*	cdA 5′*R/*5′*S*	8-oxo-Pu/cPu	Reference
S^•−^	5	249 (7.8)	4.0 (0.60)	2.5	0.65	62.3	This work
HO^•^	7	194 (7.7)	4.6 (0.60)	4.5	1.2	42.1	36

^1^ 8-oxo-Pu = 8-oxo-dG + 8-oxo-dA, and cPu = 5′*R*-cdG + 5′*S*-cdG + 5′*R*-cdA + 5′*S*-cdA.

**Table 5:** The levels (lesions/10^7^ nu/Gy) of 8-oxo-dG, 8-oxo-dA, 5′*R*-cdG, 5′*S*-cdG, 5′*R*-cdA, and 5′*S*-cdA from γ-radiolysis of N_2_-flushed aqueous solutions containing ctDNA (0.1 mg/mL) and precursor of sulfur-centered radicals. The values correspond to slopes obtained from linear regression (product formation vs. dose) and are reported as slope ± standard error (SE) ^1^.

Precursor (mM)	Reactive Species	ct-DNA mg/mL	pH	8-oxo-dG	8-oxo-dA	5′*R*-cdG	5′*S*-cdG	5′*R*-cdA	5′*S*-cdA
Na_2_S_•_9H_2_O (30)	S^•−^	0.1	5	9.31 ± 0.62	0.71 ± 0.12	0.10 ± 0.02	0.04 ± 0.01	0.09 ± 0.01	0.07 ± 0.01
HO(CH_2_)_2_SH (30)	RS^•^	0.1	7	26.76 ± 1.46	2.45 ± 0.22	0.21 ± 0.02	0.05 ± 0.01	0.22 ± 0.01	0.20 ± 0.01

^1^ Dose rate of 1.44 Gy/min.

## Data Availability

The original contributions presented in this study are included in the article/Supplementary Material. Further inquiries can be directed to the corresponding author.

## Supplementary Materials

The following supporting information can be downloaded at: https://www.mdpi.com/article/10.3390/biom16050711/s1. Figure S1A: MALDI-TOF analysis of ODN1 and ODN2, Figure S1B: UV melting curves of 10-mer duplexes, Figure S2: HPLC separation, Figure S3: ^15^*N* isotopic labeled compounds, Figure S4: Calibration curves for the quantification of the lesions, Figure S5: Radiation induced formation of lesions in dG/dA (6:4) 0.5 mg/mL, Figure S6: Radiation induced formation of lesions in ds-ODN 0.5 mg/mL, Figure S7: Radiation induced formation of lesions in ds-ODN 0.3 mg/mL, Figure S8: Radiation induced formation of lesions in ct-DNA 0.5 mg/mL, Figure S9: Radiation induced formation of lesions in ct-DNA 0.3 mg/mL, Figure S10: Radiation induced formation of lesions in ct-DNA 0.1 mg/mL, Figure S11: Radiation induced formation of lesions in ct-DNA 0.1 mg/mL using Na_2_S_•_9H_2_O (30 mM), Figure S12: Radiation induced formation of lesions in ct-DNA 0.1 mg/mL using HOCH_2_CH_2_SH (30 mM); Table S1: Sequences and molecular masses of the synthesized ODNs, Table S2: A list of MRM transitions, Table S3: Number of lesions in dG 0.5 mg/mL, Table S4: Number of lesions in dA 0.5 mg/mL, Table S5: Number of lesions in dG/dA (6:4) 0.5 mg/mL, Tables S6 and S7: Number of lesions in ds-ODN 0.5 mg/mL, Tables S8 and S9: Number of lesions in ds-ODN 0.3 mg/mL, Table S10: Number of lesions in ct-DNA 0.5 mg/mL, Table S11: Number of lesions in ct-DNA 0.3 mg/mL, Table S12: Number of lesions in ct-DNA 0.1 mg/mL, Tables S13 and S14: Number of lesions in ct-DNA 0.1 mg/mL using Na_2_S_•_9H_2_O (30 mM), Tables S15 and S16: Number of lesions in ct-DNA 0.1 mg/mL using HOCH_2_CH_2_SH (30 mM).

## Abbreviations

The following abbreviations are used in this manuscript:

cPu	purine 5′,8-cyclo-2′-deoxynucleosides
ct	calf thymus
dA	2′-deoxyadenosine
dG	2′-deoxyguanosine
ds-ODN	double-stranded oligonucleotide
ET	electron-transfer
nu	nucleosides
8-oxo-Pu	8-oxo-7,8-dihydro-2′-deoxypurine
Pu	purines
RNS	Reactive Nitrogen Species
ROS	Reactive Oxygen Species
RSS	Reactive Sulfur Species
