# Supplementary material for: Biomimetic Studies on the Reactivity of Sulfur-Centered Radicals with Purine Moieties of DNA
Source: Biomolecules. 2026 May 12;16(5):711. doi: 10.3390/biom16050711 (PMC13204028; doi:10.3390/biom16050711)
Supplement: Supplementary file 1 [file biomolecules-16-00711-s001.zip › biomolecules-4265299-supplementary.pdf]

Biomimetic Studies on the Reactivity of Sulfur-Centered Radicals with Purine Moieties of DNA

Annalisa Masi [1], Sebastian Barata-Vallejo [2,3], and Chryssostomos Chatgilialoglu [2,4,*]

[1] Istituto di Cristallografia, Consiglio Nazionale delle Ricerche, Via Salaria km 29.300, Monterotondo, 00015, Italy
[2] Istituto per la Sintesi Organica e la Fotoreattività, Consiglio Nazionale delle Ricerche, 40129 Bologna, Italy
[3] Departamento de Ciencias Químicas, Universidad de Buenos Aires, Buenos Aires CP 1113, Argentina
[4] Center for Advanced Technologies, Adam Mickiewicz University, 61-614 Poznań, Poland
* Correspondence: chrys@isof.cnr.it or chachr@amu.edu.pl

Table of contents

1.- ODNs characterization (Figures S1A-S1B, Table S1) S2-S3

2.- Enzymatic Digestion and LC-MS/MS Quantification of DNA Lesions
(Figures S2-S4, Table S2) S3-S5

3.- Dose-dependence of lesion in purine nucleosides (Figure S5, Tables S3-S5) S5-S6

4.- Dose-dependence of lesion in ds- ODNs (Figures S6-S7, Tables S6-S9) S7-S8

5.- Dose-dependence of lesion in ct-DNA (Figures S8-S12, Tables S10-S16) S9-S13

1.- ODNs characterization

Table S1. Sequences and molecular masses of the synthesized ODNs

Strands	Sequence (5'- 3')	Mass calcd (Da)	Mass[a] found (Da)
**ODN-1**	GCG TTTT GCG	3050	3048.83
**ODN-2[b]**	CGC AAAA CGC	3006	3005.30

[a]All the oligonucleotide masses were obtained by MALDI-TOF in negative mode. The mass found corresponding to $[M-1H^{+}]^{-}$ for oligonucleotides ODN1 and ODN2.
[b]Complementary strand

ODN-1 (3048.83)

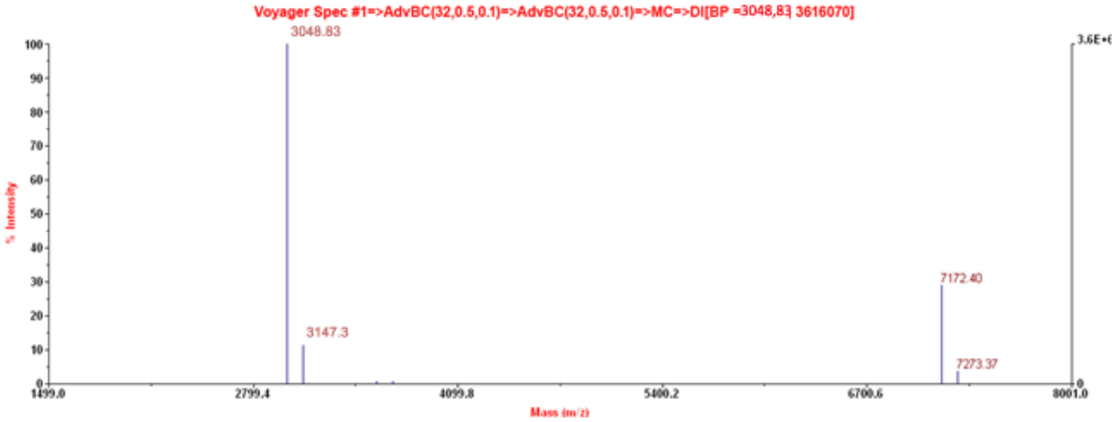

ODN-2 (3005.30)

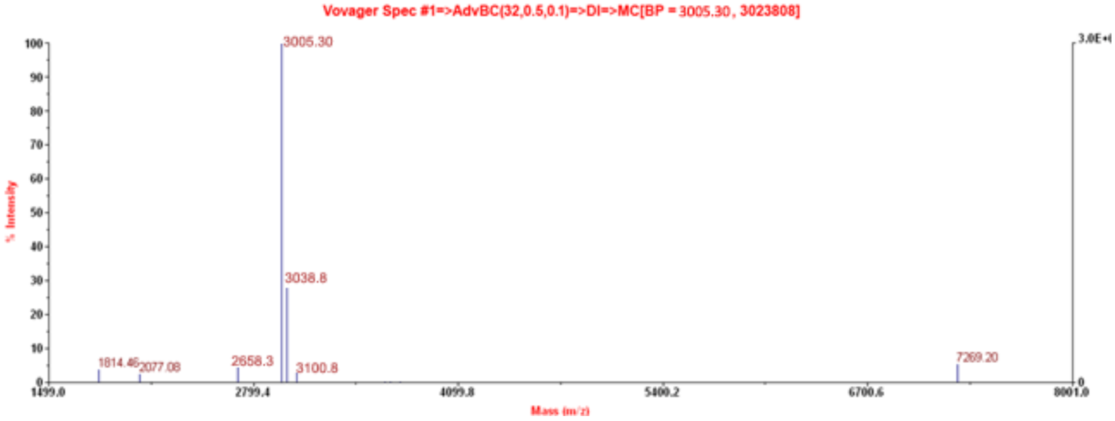

Figure S1A. MALDI-TOF analysis was performed using a Voyager DE Pro (Applied Biosystems, Foster City, CA) equipped with a pulsed N2 laser operating at 337 nm. Whole oligonucleotides negative ion spectra were acquired in linear mode over a m/z range from 2000 to 7000 using a 20000-V accelerating voltage, a 17000-V grid voltage, and a delay extraction time of 200 ns. The spectrum for each spot was obtained by averaging the result of 100 laser shots. External mass calibration was performed using peptides standard mixture (mass range: 1000-6000). The analyses were performed by spotting on the target plate 1 μL of the sample mixed with an equal volume of the matrix solution, 50 mg/mL 3-hydroxypicolinic acid/ 50 mg/mL diammonium citrate (9/1) (v/v) in water.

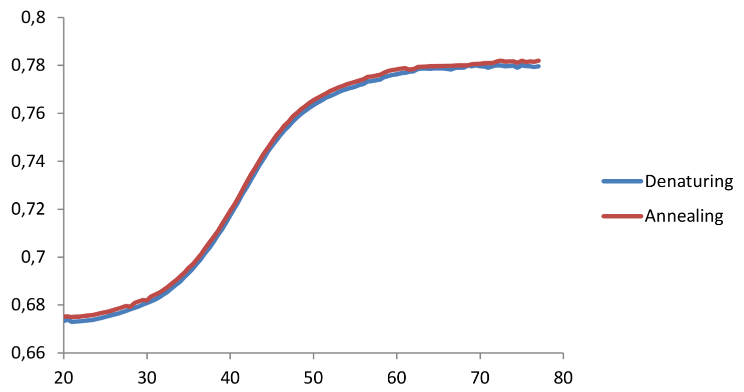

Figure S1B. UV melting curves of decamer duplexes.

2.- Enzymatic Digestion and LC-MS/MS Quantification of DNA Lesions

Enzymatic hydrolysis, HPLC clean-up/enrichment, calibration, and LC-MS/MS (MRM) analysis were performed as previously described in [1].
Detection was carried out in MRM mode using the two most intense and characteristic precursor/product ion transitions for each lesion.

1. Chatgilialoglu, C.; Krokidis, M.G.; Terzidis, M.A. Protocol for the simultaneous quantification of oxidative purine lesions in DNA using LC-MS/MS analysis. *STAR Protoc.* **2024**, *5*, 103191.

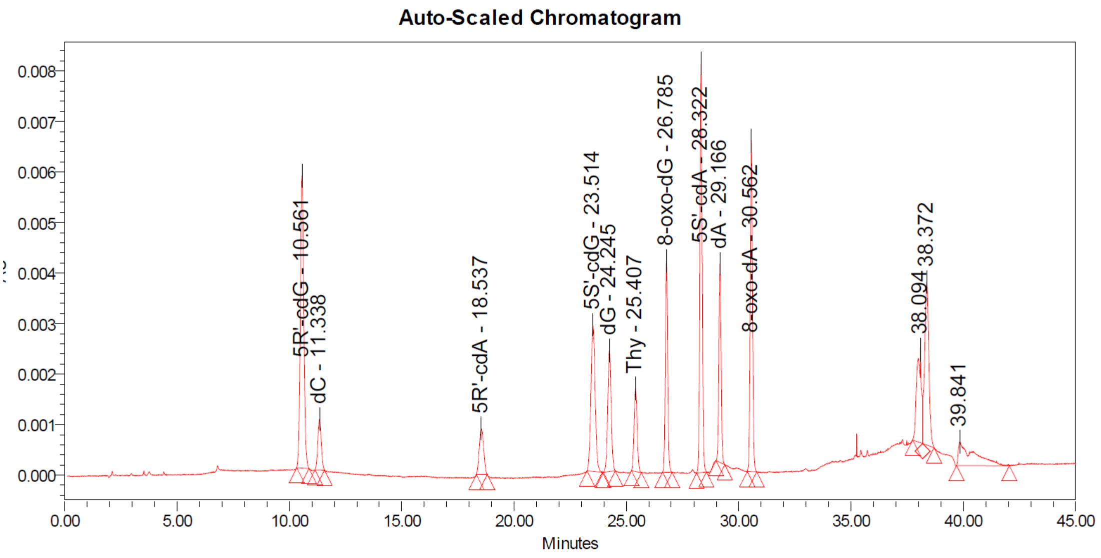

Figure S2. A representative optimal HPLC separation (monitored at 260 nm) of the 2'-deoxynucleosides (dA, dC, Thy and dG), purine 5',8-cyclo-2'-deoxynucleosides (5'*R*-cdA, 5'*S*-cdA,5'*R*-cdG and 5'*S*-cdG) and the purine 8-oxo-2'-deoxynucleosides (8-oxo-dA, 8-oxo-dG). The analysis was performed on a 4.6 mm x 150 mm Atlantis® dC18, 100 Å column (5 µm particle size, Waters) loaded with a 4.6 mm x 20 mm Guard Column 2pK (Atlantis® dC18 5µm, Waters) on a Waters Alliance ® HPLC System (Waters e2695 Separations Module) including a Waters 2998 Photodiode Array (PDA) detector; mobile phase: 2 mM ammomium formate, acetonitrile and methanol.

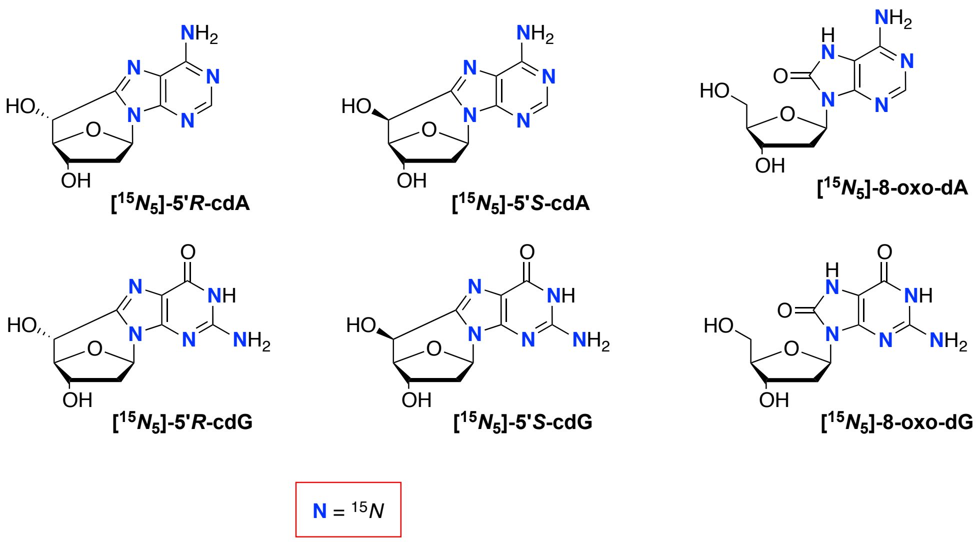

Figure S3. ^{15}N isotopically labeled compounds.

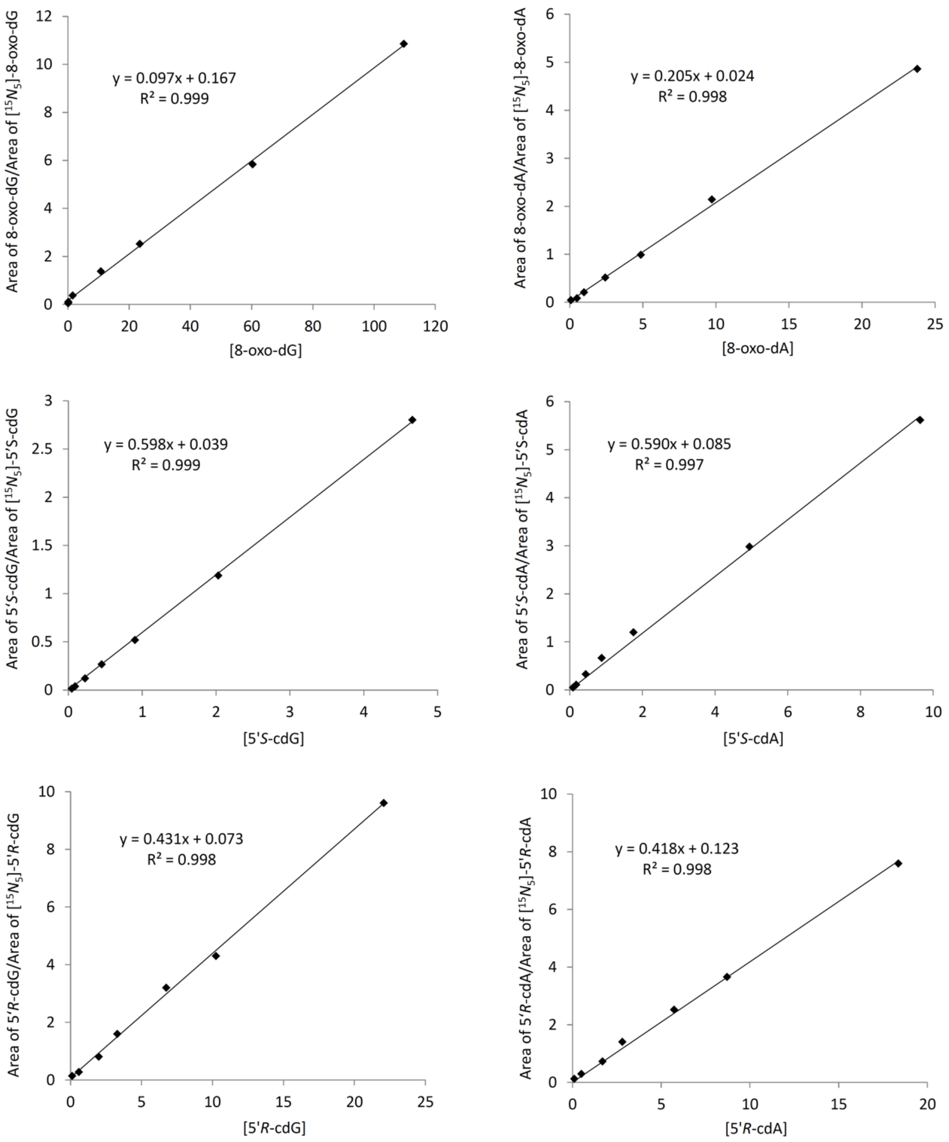

Figure S4. Calibration curves for the quantification of the lesions (nM)

Table S2. A list of MRM transitions employed for the quantification of the six oxidatively induced DNA lesions and their corresponding stable isotope-labeled standards.

	Precursor ion m/z	Product ion m/z	Collision energy (V)
5'*R*-cdA	250	164	14
[$^{15}N_5$]-5'*R*-cdA	255	169	14
5'*S*-cdA	250	164	16
[$^{15}N_5$]- 5'*S*-cdA	255	169	16
5'*R*-cdG	266	180	18
[$^{15}N_5$]- 5'*R*-cdG	271	185	18
5'*S*-cdG	266	180	16
[$^{15}N_5$]- 5'*S*-cdG	271	185	16
8-oxo-dA	267	151	19
[$^{15}N_5$]- 8-oxo-dA	272	156	19
8-oxo-dG	284	168	18
[$^{15}N_5$]- 8-oxo-dG	289	173	18

3.- Dose-dependence of lesion in purine nucleosides

Table S3. Formation of 8-oxo-dG, 5′*R*-cdG, and 5′*S*-cdG from γ-radiolysis of N_2-flushed aqueous solutions containing dG (0.5 mg/mL) and $Na_2S•9H_2O$ (0.4 M) at pH 5. The values represent the mean ± SD from n = 3 independent experiments. Data of Figure 2A.

Dose (Gy) [1]	8-oxo-dG/10^6 dG	5'*R*-cdG/10^6 dG	5'*S*-cdG/10^6 dG
0	0	0	0
10	44.91 ± 12.43	1.12 ± 0.57	0.13 ± 0.03
20	97.83 ± 18.81	2.03 ± 0.60	0.28 ± 0.07
35	127.18 ± 27.22	3.90 ± 1.27	0.53 ± 0.14

[1] Dose rate of 1.75 Gy/min

Table S4: Formation of 8-oxo-dA, 5′*R*-cdA, and 5′*S*-cdA from γ-radiolysis of N_2-flushed aqueous solutions containing dA (0.5 mg/mL) and $Na_2S•9H_2O$ (0.4 M) at pH 5. The values represent the mean ± SD from n = 3 independent experiments. Data of Figure 2B.

Dose (Gy) [1]	8-oxo-dA/10^6 dA	5'*R*-cdA/10^6 dA	5'*S*-cdA/10^6 dA
0	0	0	0
10	10.83 ± 0.70	1.20 ± 0.24	0.25 ± 0.06
20	22.82 ± 2.97	2.19 ± 0.43	0.38 ± 0.19
35	47.03 ± 10.00	4.38 ± 1.13	0.72 ± 0.08

[1] Dose rate of 1.75 Gy/min

Table S5. Formation of 8-oxo-dG, 5′*R*-cdG, 5′*S*-cdG, 8-oxo-dA, 5′*R*-cdA, and 5′*S*-cdA from γ-radiolysis of N_2-flushed aqueous solutions containing 0.5 mg/mL dG/dA ratio of 6:4 and $Na_2S \bullet 9H_2O$ (0.4 M) at pH 5. The values represent the mean ± SD from *n* = 3 independent experiments. Data of Figure S5.

Dose (Gy) [1]	8-oxo-dG/ 10^6 dG/Gy	5′*R*-cdG/ 10^6 dG/Gy	5′*S*-cdG/ 10^6 dG/Gy	8-oxo-dA/ 10^6 dA/Gy	5′*R*-cdA/ 10^6 dA/Gy	5′*S*-cdA/ 10^6 dA/Gy
0	0	0	0	0	0	0
10	29.93 ± 7.89	0.88 ± 0.47	0.11 ± 0.05	3.11 ± 1.24	0.30 ± 0.05	0.06 ± 0.00
20	65.23 ± 18.20	1.73 ± 0.82	0.25 ± 0.05	5.93 ± 1.15	0.59 ± 0.11	0.10 ± 0.05
35	88.34 ± 15.12	3.11 ± 0.76	0.48 ± 0.11	10.88 ± 2.90	1.05 ± 0.16	0.17 ± 0.03

[1] Dose rate of 1.75 Gy/min

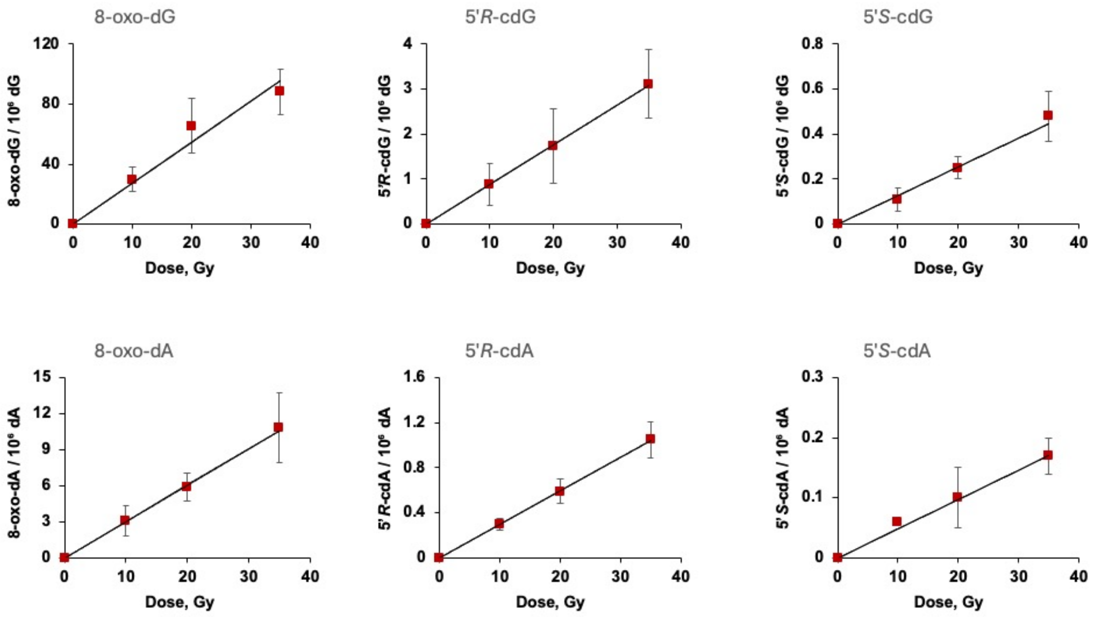

Figure S5. Formation of 8-oxo-dG, 5′*R*-cdG, 5′*S*-cdG, 8-oxo-dA, 5′*R*-cdA, and 5′*S*-cdA from γ-radiolysis of N_2-flushed aqueous solutions containing dG/dA (6:4) (1.82 mM) and $Na_2S \bullet 9H_2O$ (0.4 M) at pH 5; the samples were exposed to 10, 20, and 35 Gy doses, and the values represent the mean per 10^6 dG ± SD of n = 3 independent experiments. Dose rate of 1.75 Gy/min.

4.- Dose-dependence of lesion in ds-ODNs

Table S6. Formation of 8-oxo-dG, 8-oxo-dA, 5′*R*-cdG, 5′*S*-cdG, 5′*R*-cdA, and 5′*S*-cdA from γ-radiolysis of N_2-flushed aqueous solutions containing ds-ODN (0.5 mg/mL) and $Na_2S \cdot 9H_2O$ (0.4 M) at pH 5. The values represent the mean ± SD from *n* = 3 independent experiments. Data of Figure S6.

Dose (Gy) [1]	8-oxo-dG/ 10^6 dG/Gy	8-oxo-dA/ 10^6 dA/Gy	5′*R*-cdG/ 10^6 dG/Gy	5′*S*-cdG/ 10^6 dG/Gy	5′*R*-cdA/ 10^6 dA/Gy	5′*S*-cdA/ 10^6 dA/Gy
0	0	0	0	0	0	0
10	13.22 ± 4.27	2.18 ± 0.70	0.28 ± 0.14	0.51 ± 0.15	2.51 ± 0.31	1.98 ± 0.51
20	31.11 ± 8.52	4.69 ± 0.88	0.51 ± 0.24	1.47 ± 0.60	3.40 ± 1.03	2.46 ± 1.39
35	42.38 ± 9.10	11.24 ± 3.97	1.46 ± 0.42	2.61 ± 0.62	6.59 ± 2.28	5.76 ± 0.92

[1] Dose rate of 1.75 Gy/min

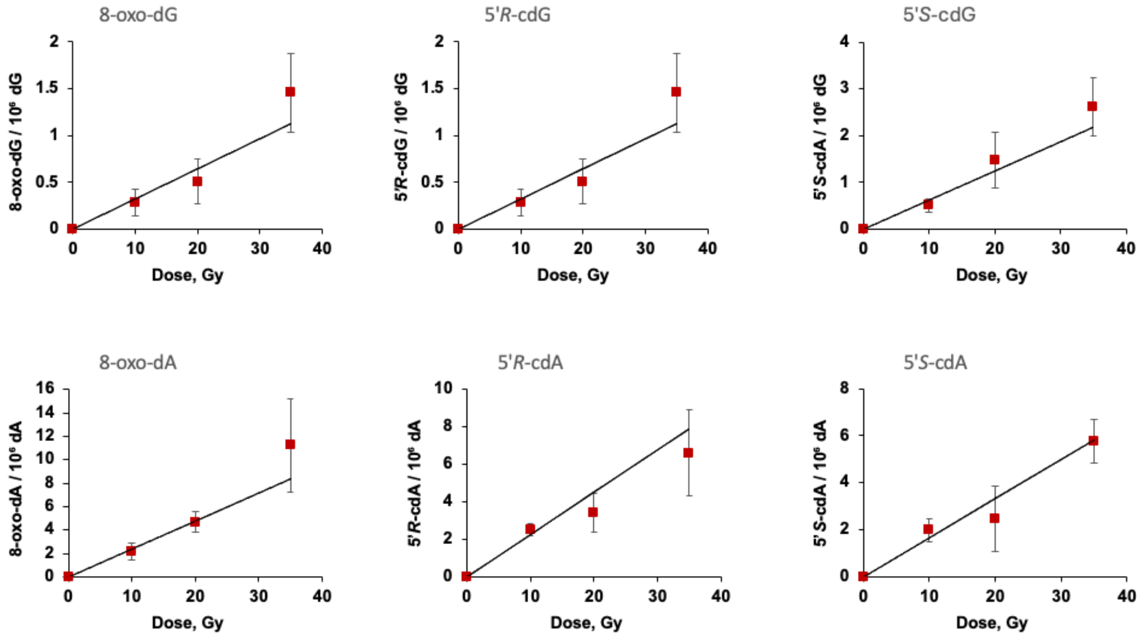

Figure S6. Formation of 8-oxo-dG, 5′*R*-cdG, 5′*S*-cdG, 8-oxo-dA, 5′*R*-cdA, and 5′*S*-cdA from γ-radiolysis of N_2-flushed aqueous solutions containing ds-ODN (0.5 mg/mL) and $Na_2S \cdot 9H_2O$ (0.4 M) at pH 5; the samples were exposed to 10, 20, and 35 Gy doses, and the values represent the mean ± SD of n = 3 independent experiments. Dose rate of 1.75 Gy/min.

Table S7. Formation of 8-oxo-Pu and cPu from γ-radiolysis of N_2-flushed aqueous solutions containing ds-ODN (0.5 mg/mL) and $Na_2S \cdot 9H_2O$ (0.4 M) at pH 5. The values represent the mean ± SD from *n* = 3 independent experiments. Data of Figure 4A.

Dose (Gy) [1]	8-oxo-Pu/10^6 nu	cPu/10^6 nu
0	0	0
10	15.97 ± 7.54	5.28 ± 0.94
20	35.80 ± 9.31	7.83 ± 2.11
35	53.61 ± 6.92	12.22 ± 3.55

[1] Dose rate of 1.75 Gy/min

Table S8. Formation of 8-oxo-dG, 8-oxo-dA, 5′*R*-cdG, 5′*S*-cdG, 5′*R*-cdA, and 5′*S*-cdA from γ-radiolysis of N_2-flushed aqueous solutions containing ds-ODN (0.3 mg/mL) and Na_2S•$9H_2O$ (0.4 M) at pH 5. The values represent the mean ± SD from *n* = 3 independent experiments. Data of Figure S7.

Dose (Gy) [1]	8-oxo-dG/ 10^6 dG/Gy	8-oxo-dA/ 10^6 dA/Gy	5′*R*-cdG/ 10^6 dG/Gy	5′*S*-cdG/ 10^6 dG/Gy	5′*R*-cdA/ 10^6 dA/Gy	5′*S*-cdA/ 10^6 dA/Gy
0	0	0	0	0	0	0
10	8.35 ± 3.06	1.35 ± 0.46	0.19 ± 0.09	0.48 ± 0.10	1.51 ± 0.18	0.76 ± 0.15
20	23.97 ± 4.01	3.10 ± 0.21	0.46 ± 0.19	0.88 ± 0.45	2.19 ± 0.94	1.58 ± 0.68
35	27.59 ± 13.79	6.39 ± 1.68	1.02 ± 0.17	2.38 ± 0.41	3.95 ± 1.21	4.86 ± 0.80

[1] Dose rate of 1.75 Gy/min

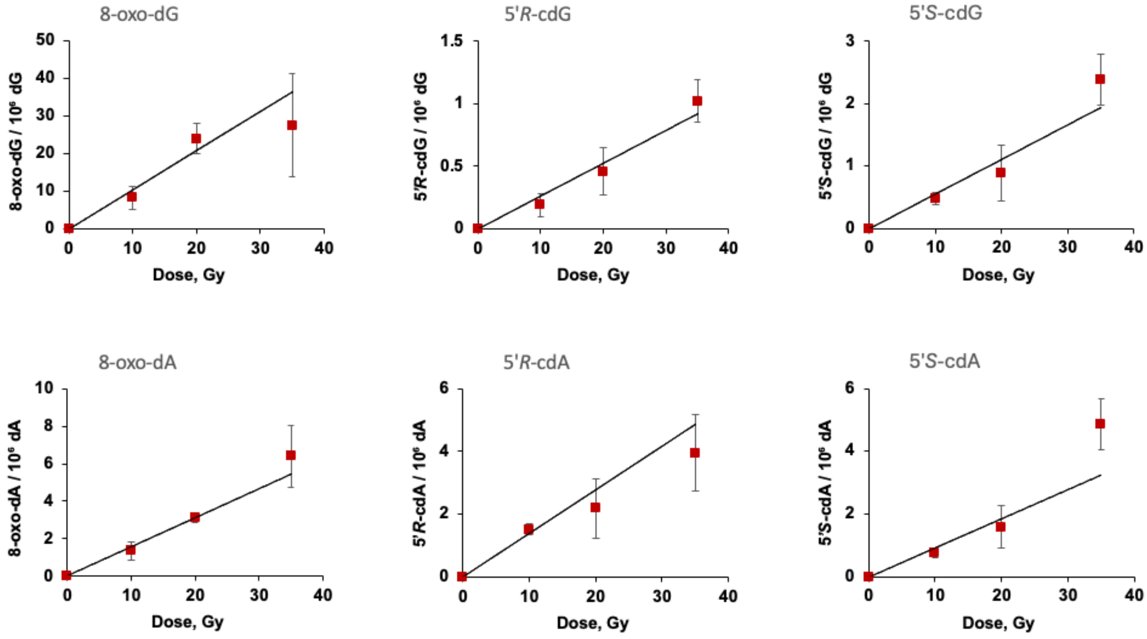

Figure S7. Formation of 8-oxo-dG, 5′*R*-cdG, 5′*S*-cdG, 8-oxo-dA, 5′*R*-cdA, and 5′*S*-cdA from γ-radiolysis of N_2-flushed aqueous solutions containing ds-ODN (0.3 mg/mL) and Na_2S•$9H_2O$ (0.4 M) at pH 5. The values represent the mean ± SD of n = 3 independent experiments. Dose rate of 1.75 Gy/min.

Table S9. Formation of 8-oxo-Pu and cPu from γ-radiolysis of N_2-flushed aqueous solutions containing ds-ODN (0.3 mg/mL) and Na_2S•$9H_2O$ (0.4 M) at pH 5. The values represent the mean ± SD from *n* = 3 independent experiments. Data of Figure 4B.

Dose (Gy) [1]	8-oxo-Pu/10^6 nu	cPu/10^6 nu
0	0	0
10	9.35 ± 3.51	2.94 ± 0.24
20	27.07 ± 4.20	5.12 ± 1.37
35	33.98 ± 13.32	12.21 ± 1.39

[1] Dose rate of 1.75 Gy/min

5.- Dose-dependence of lesion in ct-DNA

Table S10. Formation of 8-oxo-dG, 8-oxo-dA, 5′*R*-cdG, 5′*S*-cdG, 5′*R*-cdA, and 5′*S*-cdA from γ-radiolysis of N_2-flushed aqueous solutions containing ct-DNA (0.5 mg/mL) and $Na_2S{\bullet}9H_2O$ (0.4 M) at pH 5. The values represent the mean ± SD from $n = 3$ independent experiments (for 0 Gy single run). Data of Figure S8.

Dose, Gy [1]	8-oxo-dG	8-oxo-dA	5'*R*-cdG	5'*S*-cdG	5'*R*-cdA	5'*S*-cdA
**0**	2.62	0.50	0.04	0.03	0.06	0.08
**10**	193 ± 70.04	21.06 ± 7.08	0.94 ± 0.20	0.44 ± 0.10	0.98 ± 0.38	1.41 ± 0.64
**20**	461.17 ± 212.14	57.63 ± 20.56	2.25 ± 0.53	0.89 ± 0.22	1.85 ± 0.68	2.95 ± 0.78
**35**	885.83 ± 286.91	107.30 ± 14.67	5.01 ± 1.30	1.46 ± 0.38	3.59 ± 0.75	5.50 ± 0.82

[1] Dose rate of 1.87 Gy/min

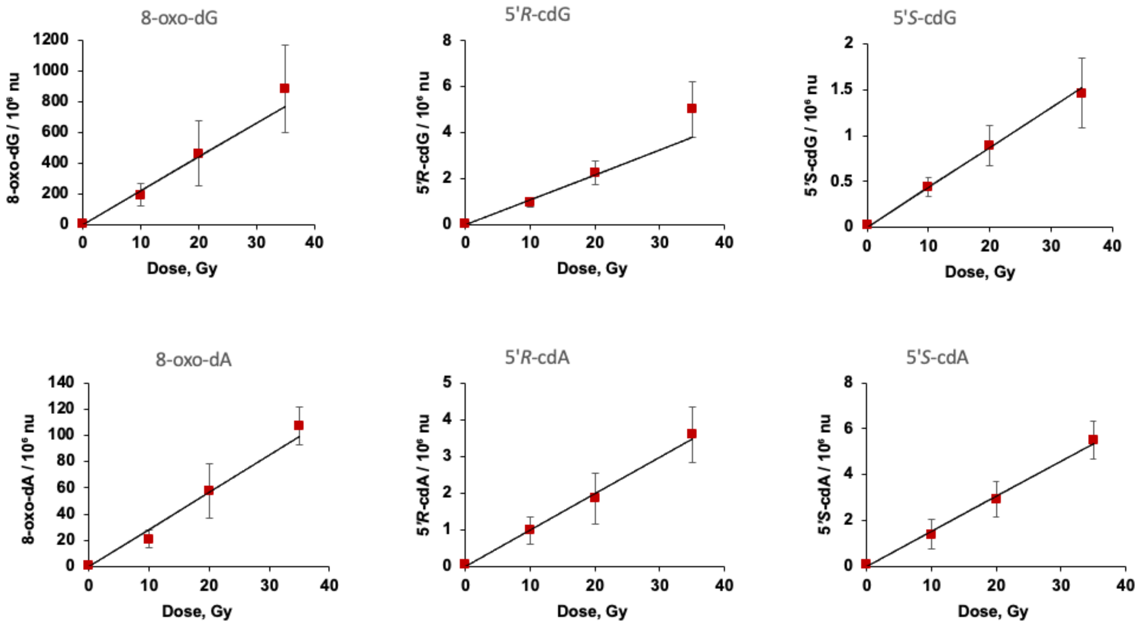

Figure S8. Formation of 8-oxo-dG, 8-oxo-dA, 5′*R*-cdG, 5′*S*-cdG, 5′*R*-cdA, and 5′*S*-cdA from γ-radiolysis of N_2-flushed aqueous solutions containing ct-DNA (0.5 mg/mL) and $Na_2S{\bullet}9H_2O$ (0.4 M) at pH 5. The values represent the mean ± SD from $n = 3$ independent experiments (for 0 Gy single run).

Table S11. Formation of 8-oxo-dG, 8-oxo-dA, 5′*R*-cdG, 5′*S*-cdG, 5′*R*-cdA, and 5′*S*-cdA from γ-radiolysis of N_2-flushed aqueous solutions containing ct-DNA (0.3 mg/mL) and $Na_2S{\bullet}9H_2O$ (0.4 M) at pH 5. The values represent the mean ± SD from $n = 3$ independent experiments (for 0 Gy single run). Data of Figure S9.

Dose, Gy [1]	8-oxo-dG	8-oxo-dA	5'*R*-cdG	5'*S*-cdG	5'*R*-cdA	5'*S*-cdA
**0**	1.05	0.25	0.04	0.02	0.05	0.05
**10**	80.90 ± 38.64	11.16 ± 3.20	0.50 ± 0.10	0.22 ± 0.07	0.56 ± 0.10	0.77 ± 0.36
**20**	215.53 ± 106.82	27.90 ± 10.69	1.09 ± 0.38	0.42 ± 0.09	0.89 ± 0.32	1.44 ± 0.32
**35**	439.65 ± 144.07	53.56 ± 7.05	2.36 ± 0.61	0.76 ± 0.19	1.78 ± 0.34	2.74 ± 0.39

[1] Dose rate of 1.87 Gy/min

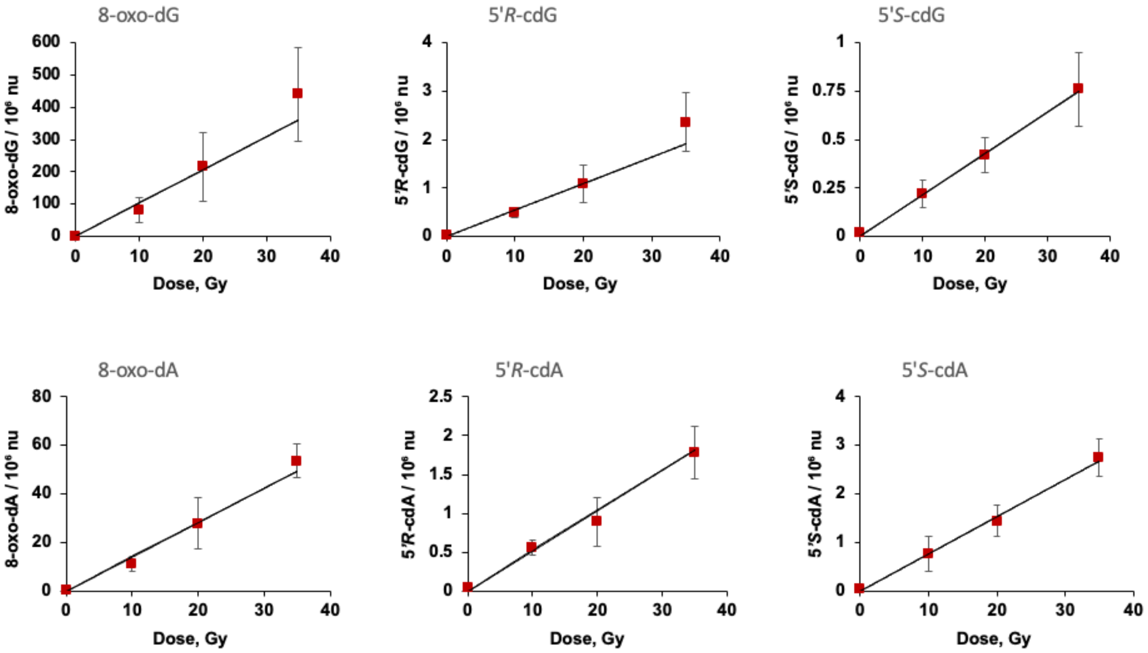

Figure S9. Formation of 8-oxo-dG, 8-oxo-dA, 5′*R*-cdG, 5′*S*-cdG, 5′*R*-cdA, and 5′*S*-cdA from γ-radiolysis of N_2-flushed aqueous solutions containing ct-DNA (0.3 mg/mL) and $Na_2S \cdot 9H_2O$ (0.4 M) at pH 5. The values represent the mean ± SD from n = 3 independent experiments (for 0 Gy single run).

Table S12. Formation of 8-oxo-dG, 8-oxo-dA, 5′*R*-cdG, 5′*S*-cdG, 5′*R*-cdA, and 5′*S*-cdA from γ-radiolysis of N_2-flushed aqueous solutions containing ct-DNA (0.1 mg/mL) and $Na_2S \cdot 9H_2O$ (0.4 M) at pH 5. The values represent the mean ± SD from n = 3 independent experiments (for 0 Gy single run). Data of Figure S10.

**Dose, Gy** [1]	**8-oxo-dG**	**8-oxo-dA**	**5'*R*-cdG**	**5'*S*-cdG**	**5'*R*-cdA**	**5'*S*-cdA**
**0**	0.44	0.11	0.04	0.02	0.05	0.03
**10**	36.52 ± 11.70	3.95 ± 1.34	0.20 ± 0.03	0.10 ± 0.03	0.22 ± 0.07	0.29 ± 0.14
**20**	91.27 ± 39.80	10.97 ± 3.66	0.46 ± 0.09	0.19 ± 0.04	0.40 ± 0.13	0.57 ± 0.11
**35**	160.89 ± 58.92	20.24 ± 3.83	0.96 ± 0.31	0.28 ± 0.07	0.69 ± 0.11	1.01 ± 0.17

[1] Dose rate of 1.87 Gy/min

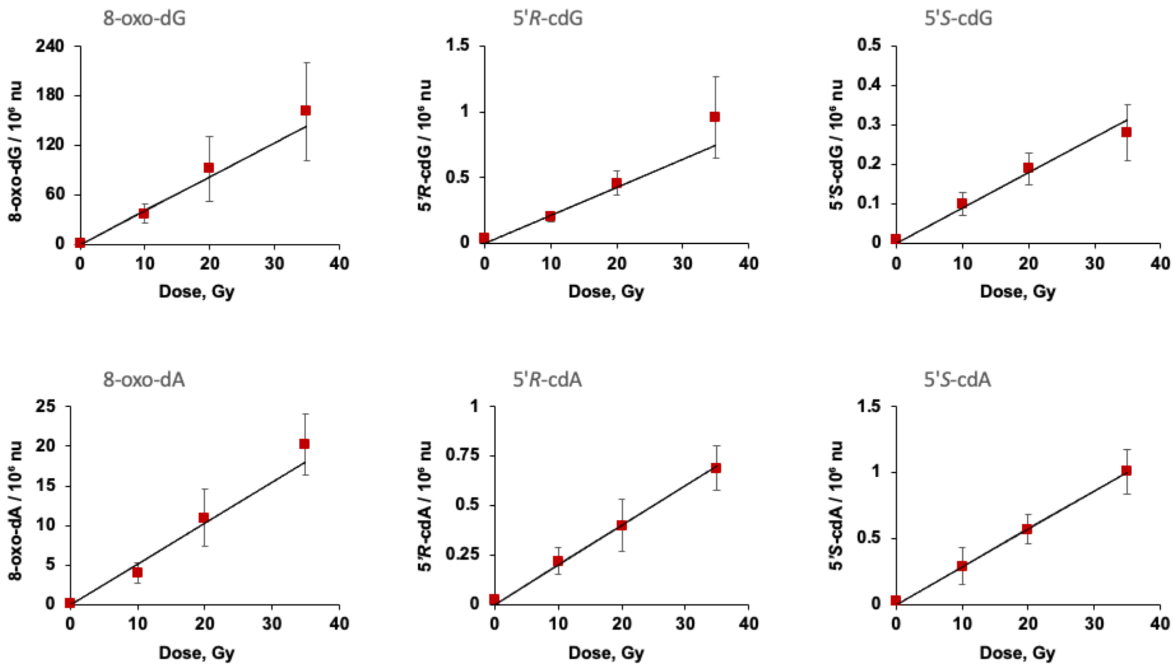

Figure S10. Formation of 8-oxo-dG, 8-oxo-dA, 5′*R*-cdG, 5′*S*-cdG, 5′*R*-cdA, and 5′*S*-cdA from γ-radiolysis of N_2-flushed aqueous solutions containing ct-DNA (0.1 mg/mL) and $Na_2S{\bullet}9H_2O$ (0.4 M) at pH 5. The values represent the mean ± SD from *n* = 3 independent experiments (for 0 Gy single run).

Table S13. The levels (lesions/10^6 nucleosides) of 8-oxo-dG, 8-oxo-dA, 5'*R*-cdG, 5'*S*-cdG, 5'*R*-cdA, and 5'*S*-cdA, in irradiated ct-DNA (0.1 mg/mL) samples in the presence of $Na_2S{\bullet}9H_2O$ (30 mM) at pH 5 (pH adjusted with H_3PO_4). The values represent the mean ± SD of n = 3 independent experiments (0 Gy sample is duplicated). Data of Figure S11.

Dose, Gy [1]	8-oxo-dG	8-oxo-dA	5'*R*-cdG	5'*S*-cdG	5'*R*-cdA	5'*S*-cdA
0	0.14 ± 0.03	0	0	0	0	0
10	8.33 ± 1.20	0.82 ± 0.05	0.07 ± 0.01	0.03 ± 0.00	0.12 ± 0.03	0.05 ± 0.01
20	21.12 ± 2.51	1.15 ± 0.03	0.23 ± 0.02	0.10 ± 0.02	0.21 ± 0.02	0.17 ± 0.02
35	32.16 ± 3.74	3.31 ± 0.07	0.44 ± 0.04	0.11 ± 0.02	0.29 ± 0.02	0.24 ± 0.03

[1] Dose rate of 1.44 Gy/min.

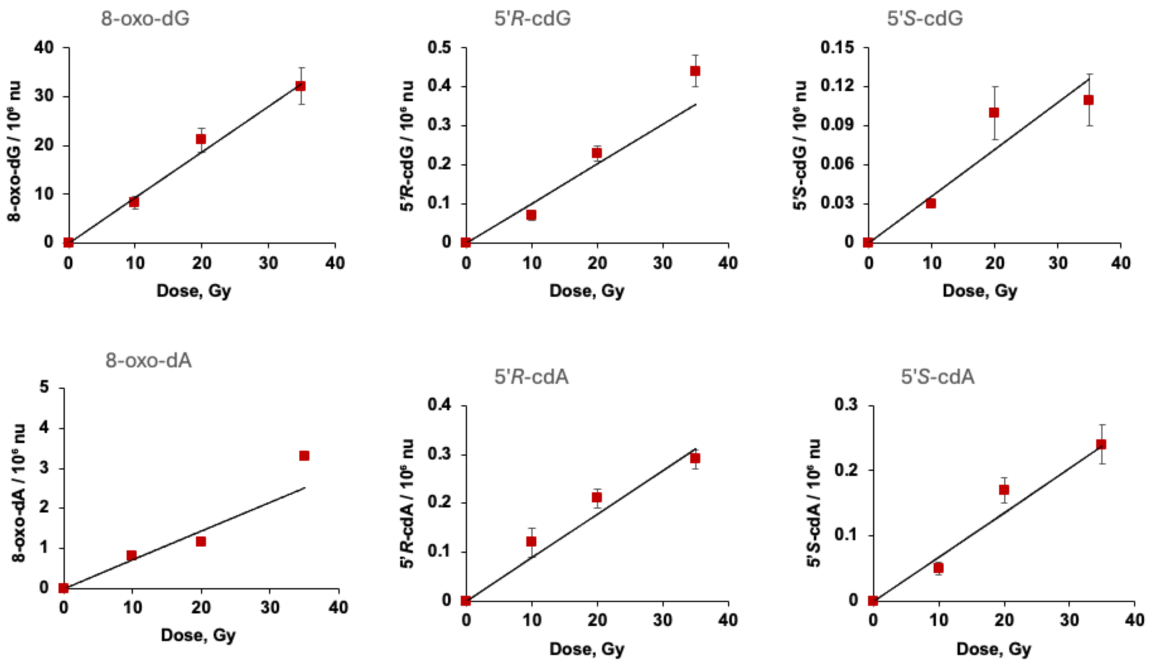

Figure S11. Formation of 8-oxo-dG, 5′*R*-cdG, 5′*S*-cdG, 8-oxo-dA, 5′*R*-cdA, and 5′*S*-cdA from γ-radiolysis of N_2-flushed aqueous solutions containing ct-DNA (0.1 mg/mL) and $Na_2S•9H_2O$ (30 mM) at pH 5 (pH adjusted with H_3PO_4). The samples were exposed to 10, 20, and 35 Gy doses. The values represent the mean per 10^6 nu ± SD, based on n = 3 independent experiments. Dose rate of 1.44 Gy/min.

Table S14. Total amount of 8-oxo-Pu and cPu lesions (lesions/10^6 nucleosides) in irradiated ct-DNA (0.1 mg/mL) samples in the presence of $Na_2S•9H_2O$ (30 mM) at pH 5 (pH adjusted with H_3PO_4). The values represent the mean ± SD of n = 3 independent experiments (0 Gy sample is duplicated). Data of Figure 6A.

Dose, Gy [1]	8-oxo-Pu/10^6 nu	cPu/10^6 nu
0	0.14 ± 0.03	0
10	9.15 ± 0.26	0.27 ± 0.01
20	22.28 ± 2.50	0.71 ± 0.03
35	35.47 ± 3.68	1.08 ± 0.06

[1] Dose rate of 1.44 Gy/min.

Table S15. The levels (lesions/10^6 nucleosides) of 8-oxo-dG, 8-oxo-dA, 5'*R*-cdG, 5'*S*-cdG, 5'*R*-cdA, and 5'*S*-cdA, in irradiated ct-DNA (0.1 mg/mL) samples in the presence of 2-mercaptoethanol (30 mM) at natural pH. The values represent the mean ± SD of n = 3 independent experiments (0 Gy sample is duplicated). Data of Figure S12.

Dose (Gy) [1]	8-oxo-dG	8-oxo-dA	5'*R*-cdG	5'*S*-cdG	5'*R*-cdA	5'*S*-cdA
0	0.38 ± 0.08	0	0	0	0	0
10	25.30 ± 2.20	2.03 ± 0.13	0.17 ± 0.02	0.06 ± 0.00	0.22 ± 0.05	0.21 ± 0.04
20	62.32 ± 7.40	4.61 ± 0.14	0.39 ± 0.03	0.09 ± 0.01	0.42 ± 0.04	0.39 ± 0.04
35	93.19 ± 8.92	10.03 ± 0.31	0.88 ± 0.06	0.16 ± 0.02	0.83 ± 0.06	0.73 ± 0.09

[1] Dose rate of 1.44 Gy/min.

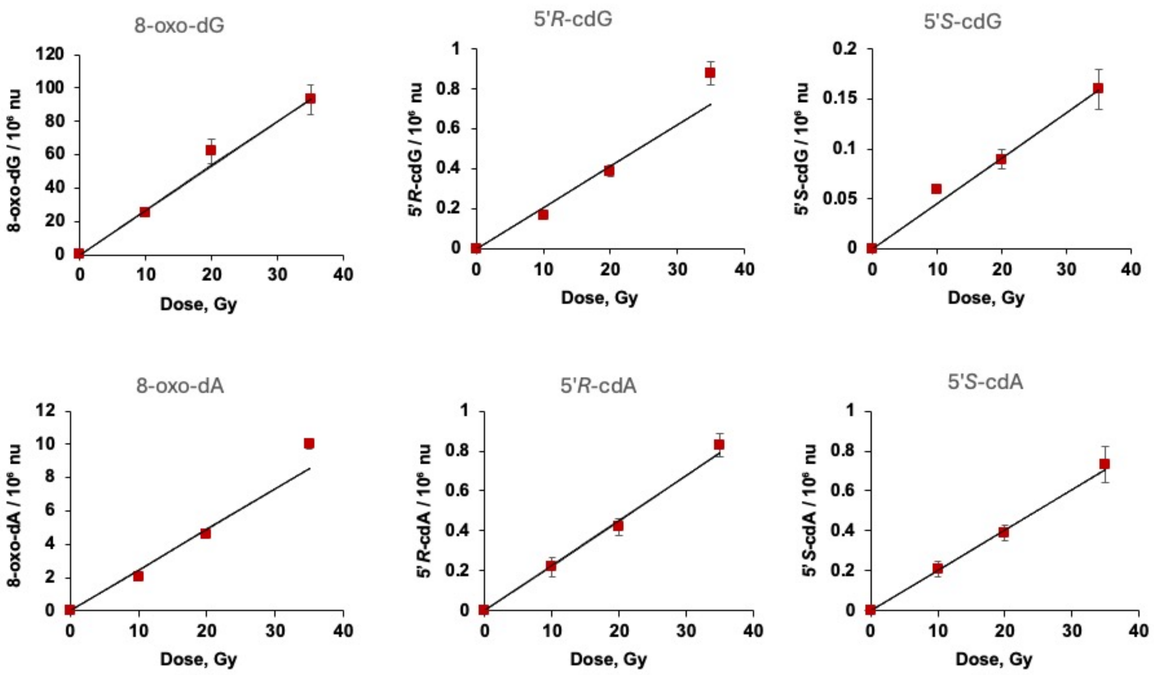

Figure S12. Formation of 8-oxo-dG, 5′*R*-cdG, 5′*S*-cdG, 8-oxo-dA, 5′*R*-cdA, and 5′*S*-cdA from γ-radiolysis of N_2-flushed aqueous solutions containing ct-DNA (0.1 mg/mL) and $HOCH_2CH_2SH$ (30 m M) at pH 7 (natural pH). The samples were exposed to 10, 20, and 35 Gy doses. The values represent the mean per 10^6 nu ± SD, based on n = 3 independent experiments. Dose rate of 1.44 Gy/min.

Table S16. Total amount of cPu and 8-oxo-Pu lesions (lesions/10^6 nucleosides) in irradiated ct-DNA (0.1 mg/mL) samples in the presence of 2-mercaptoethanol (30 mM) at pH 7 (natural pH). The values represent the mean ± SD of n = 3 independent experiments (0 Gy sample is duplicated). Data of Figure 6B.

Dose (Gy) [1]	8-oxo-Pu/10^6 nu	cPu/10^6 nu
0	0.38 ± 0.08	0
10	27.34 ± 2.28	0.66 ± 0.06
20	66.93 ± 7.47	1.29 ± 0.07
35	103.22 ± 8.81	2.60 ± 0.11

[1] Dose rate of 1.44 Gy/min.
